# Mendelian randomization and transcriptome analysis reveal depression-driven regulatory patterns of the immune microenvironment in myocardial infarction and heart failure

**DOI:** 10.3389/fimmu.2026.1727699

**Published:** 2026-01-29

**Authors:** Zihao Zhou, Xiaotongning Yu, Yani Jin, Ziyi Liu, Guoqiang Liang, Ben Ma, Anqi Hou, Tongfei Gu, Na Xu, Shuo Sun

**Affiliations:** 1 School of Life Sciences, Jining Medical University, Rizhao, China; 2 School of Modern Chinese Medicine Industry, Chengdu University of Traditional Chinese Medicine, Chengdu, China; 3 Department of Cardiology, Sir Run Run Shaw Hospital, School of Medicine, Zhejiang University, Hangzhou, China

**Keywords:** cardiovascular disease, immune microenvironment, major depressive disorder, mendelian randomization, transcriptomics

## Abstract

**Background:**

Major depressive disorder (MDD) and cardiovascular diseases (CVD) are mutually amplifying global health burdens, yet the causal directions and immune-determined molecular substructures that link MDD to myocardial infarction (MI) and heart failure (HF) remain poorly resolved.

**Methods:**

Bidirectional two-sample Mendelian randomization (MR) was applied to large-scale GWAS (1.35 million MDD; 361 K MI; 977 K HF) followed by replication in 11,004 NHANES 2005–2020 participants using restricted cubic splines and multivariable logistic regression. Multi-cohort transcriptomics (peripheral blood microarray n = 447; in-house RNA-seq n = 14; left-ventricular tissue from dilated cardiomyopathy (DCM) patients (n = 332) were integrated to identify MDD-driven expression signatures. LASSO regression, CIBERSORT, ssGSEA, consensus clustering and GSVA were employed to derive diagnostic gene panels and immune endotypes.

**Results:**

MR analyses provided genetic evidence consistent with a directional effect of MDD on MI (IVW β = 0.01, P = 4.6 × 10⁻^6^) and HF (IVW β = 0.19, P = 1.3 × 10⁻^6^) without reverse causation. Depression (PHQ-9 ≥ 10) has a dose-dependent nonlinear association with MI and HF (P<0.0001), with adjusted odds ratios (OR) of 1.80 (95% CI: 1.07-3.05) and 2.41 (95% CI: 1.45-4.00), respectively. A total of 202 MDD-related genes were identified through integrated transcriptomic analysis. After cross validation with the MI/HF dataset, six robust biomarkers (*TMEM43*, *C1orf174*, *L3MBTL4*, *OR52N4*, *SLC25A20*, *MISP3*) were screened. Risk-score models discriminated MI (AUC = 0.90–1.00) and HF (AUC = 0.95) in peripheral blood, but HF discrimination in cardiac tissue was modest (AUC = 0.60). Consensus clustering on 184 MDD-correlated genes stratified each CVD into two reproducible subtypes: a “homeostatic/pro-fibrotic” cluster enriched for ribosomal and cell-cycle pathways and an “inflammatory-metabolic” cluster characterized by NF-κB, TNF-α, IL-6, complement and coagulation activation.

**Conclusions:**

Genetic, epidemiological, and multi-omic evidence supports a directional association between MDD and increased risk of MI and HF. We deliver reproducible blood-based gene panels and immune endotypes that dissect biologically distinct MDD-CVD substructures, offering actionable targets for precision immunomodulatory therapy in cardio-depressive comorbidity.

## 1 Introduction

Cardiovascular diseases (CVD) and mental health disorders (MHD) are among the leading causes of mortality and disability worldwide, consistently occupying a central position in the global disease spectrum (1, 2). In recent years, growing evidence has shown a high comorbidity between CVD and MHD, with shared genetic susceptibilities and environmental exposures. Moreover, these conditions may interact through mechanisms such as neuroendocrine regulation, inflammatory responses, and lifestyle changes (3). CVD encompasses various clinical conditions, primarily including hypertension, coronary artery disease (CAD), heart failure (HF), myocardial infarction (MI), stroke, and atrial fibrillation. Among these, HF and MI are two major subtypes, both exhibiting high prevalence and mortality, posing serious threats to human health (1, 2). MHD represents a highly heterogeneous group of neuropsychiatric disorders, including major depressive disorder (MDD), bipolar disorder (BD), schizophrenia (SCZ), and autism spectrum disorder (ASD) (3). Cohort analyses from the All of Us project have demonstrated that MDD and BD significantly increase the risk of future HF and composite CVD events, particularly in female patients (4). Additionally, behavioral cardiology reviews have noted that the comorbidity of CVD and MHD not only adversely affects prognosis but also increases the overall health burden (5). Among MHD, MDD is one of the most common and disabling disorders, affecting approximately 5% of adults globally, and significantly impairing quality of life and social functioning (6, 7). Numerous studies have reported a higher risk of MHD in patients with CVD (8), with prevalence varying according to the impact of CVD on life expectancy and quality of life (5). Upcoming cardiac surgery is also an important trigger for certain cognitive and emotional responses in CVD patients (9).

Studies investigating the causal direction between MHD and CVD have yielded inconsistent conclusions across clinical, cohort, and Mendelian randomization (MR) studies. The prevalence of MHD is higher in CVD patients than in the general population, leading some researchers to hypothesize that CVD may causally contribute to MHD development (5). Cohort studies have indicated a positive association between MHD and CVD after adjusting for demographic factors (e.g., age, race, sex, and education) and potential risk factors (e.g., smoking, alcohol consumption, dyslipidemia, and hypertension) (10). Interestingly, some MR studies suggest a unidirectional causal effect of genetic MHD on genetic CVD, with no evidence of reverse causation (10–12). However, most previous MR studies focused on a single MHD (e.g., MDD) and generalized CVD phenotypes, lacking systematic causal verification of multiple MHD subtypes (e.g., BD, SCZ) on key CVD subtypes (MI and HF). Additionally, clinical and cohort studies cannot completely rule out confounding factors such as occult inflammation and medication interference, making the causal direction controversial and unresolved.

The comorbidity mechanisms of MHD and CVD are complex and remain under active investigation. Some studies propose that autonomic nervous system dysfunction represents a shared factor. In genetically predisposed individuals, CVD-induced autonomic imbalance may trigger MHD onset (5). Transcriptomic studies have shown that, compared to controls, MHD patients exhibit upregulation of TNF-α and IL-6 signaling pathways and a prothrombotic state. These proinflammatory abnormalities may promote atherosclerotic lesions and exacerbate endothelial dysfunction (5). Postoperative MHD may be related to inflammatory processes, such as elevated levels of proinflammatory cytokines inducing neuroinflammation, as well as altered cytochrome C-cardiolipin peroxidase function and hypothalamic-pituitary-adrenal (HPA) axis dysregulation, which can enhance stress and cortisol responses (13, 14). Moreover, immune-inflammatory responses triggered by MI and MHD can interact, contributing to disease onset or progression. For instance, MI-activated immune responses may alter blood-brain barrier permeability, inducing neuroinflammation and potentially leading to MDD (15). Notably, existing mechanistic studies are mostly single-dimensional, failing to integrate immune microenvironment characteristics (e.g., immune cell infiltration in peripheral blood and cardiac tissue) with classical pathways. Moreover, the specific molecular chains by which MHD-related genes regulate immune-metabolic pathways to affect CVD progression remain unclear, and there is a lack of systematic analysis of heterogeneous mechanisms underlying different MHD-driven CVD. Furthermore, few studies have explored molecular stratification of CVD based on MHD-related genes, which limits the understanding of individual differences in MHD susceptibility among CVD patients. Subtype classification plays an important role in revealing underlying biological differences and guiding individualized therapy. This approach has been widely applied in comorbidity research, though only a few studies have proposed stratifying CVD based on key MHD genes (16). Selecting appropriate MHD-related genes for CVD stratification may hold significant future value.

Given the ongoing debate regarding the causal relationship and molecular mechanisms between MHD and CVD, and the aforementioned research gaps (i.e., lack of systematic causal verification of multiple MHD-CVD subtypes, insufficient exploration of immune microenvironment-mediated mechanisms, and absence of gene-based CVD molecular stratification), this study systematically explores these associations using multi-dimensional data and approaches. Leveraging large-scale genome-wide association study (GWAS) data, we performed MR analyses to investigate the causal effects of six MHD on MI and HF, which fills the gap of previous MR studies focusing on single MHD and generalized CVD phenotypes, and combined these with cohort analysis from the U.S. National Health and Nutrition Examination Survey (NHANES) to evaluate MDD-CVD associations. We further analyzed multiple peripheral blood and cardiac tissue transcriptomic datasets to perform differential expression analysis, least absolute shrinkage and selection operator (LASSO) feature selection, and immune infiltration assessment, identifying MDD-associated key CVD risk genes. This integrates immune microenvironment analysis with transcriptomic profiling, addressing the lack of multi-dimensional mechanistic exploration in previous studies. Cluster analysis revealed two MDD-related expression patterns in CVD patients, and gene set variation analysis (GSVA) along with functional enrichment analyses elucidated the heterogeneous mechanisms of immune regulation, metabolism, and inflammation. Different from previous studies that lacked molecular stratification, this study first realizes gene-based stratification of CVD patients related to MHD, providing a basis for personalized diagnosis and intervention.

## 2 Materials and methods

### 2.1 MR analysis of MHD and CVD

This study utilized publicly available summary-level datasets from the Psychiatric Genomics Consortium (PGC) and other consortia. We collected GWAS datasets on six MHD and two CVD. The six MHD included Alzheimer’s disease (AD) (n = 457,811), AD (n = 46,351), BD (n = 352,006), MDD (n = 1,345,483), Parkinson’s disease (PD) (n = 482,730), and SCZ (n = 320,404). The two CVD included MI (n = 361,194) and HF (n = 977,323). All datasets included only patients of European ancestry (**Supplementary Table S1**). The analysis process is presented in **Supplementary Figure S1**.

MR analyses were conducted within the TwoSampleMR framework (V0.6.6) using two models. The primary method was the inverse-variance weighting (IVW) model (17), supplemented by the MR-Egger model. The intercept obtained from MR-Egger regression was used to assess the presence of horizontal pleiotropy (18). Heterogeneity was evaluated by Cochran’s Q test (*p* < 0.05 indicating significant heterogeneity) (19). In addition to the IVW and MR-Egger models, we performed a set of complementary sensitivity analyses, including the weighted median, weighted mode, and MR-PRESSO (Global Test and Outlier Test) to evaluate the robustness of causal estimates and detect potential horizontal pleiotropy. For MR-PRESSO, outlier SNPs were removed when detected, and causal estimates were recalculated using the outlier-corrected models. We applied false discovery rate (FDR) correction using the Benjamini–Hochberg procedure. Significant associations were determined based on p-values from the IVW model. In the exposure datasets, single nucleotide polymorphisms (SNPs) reaching genome-wide significance (*p* < 5 × 10^-8^) were selected as instrumental variables (IVs), with linkage disequilibrium thresholds set at r² < 0.001 within a 10,000 kb window. Weak IVs (F > 10) were excluded to obtain independent IVs. Reverse MR analysis is performed on the disease of interest to explore its reverse causal relationship.

The primary endpoints were predefined as the causal effects of MDD on MI and HF, based on prior epidemiological and mechanistic evidence. MR analyses of the remaining psychiatric disorders (AD, ASD, BD, PD, SCZ) were treated as secondary exploratory endpoints. Effect estimates were reported as beta coefficients (β) or odds ratios (OR), representing the causal effect on the outcome per unit increase in the log-odds of genetic liability to the exposure.

### 2.2 NHANES-based cohort study of depression and CVD

The NHANES is a nationwide survey designed to collect comprehensive data on the nutrition and health status of the U.S. population. The dataset is publicly available and collected under ethical standards, including informed consent from all participants. Detailed descriptions of the study design and NHANES data are available at: “www.cdc.gov/nchs/nhanes/”. All procedures adhered strictly to ethical guidelines and regulations. We analyzed NHANES datasets from 2005 to 2020, initially including 76,496 participants. Missing values in demographic and behavioral covariates were handled using listwise deletion; no imputation was performed. Exclusions were applied as follows: 33,084 participants under 20 years old, 4,433 participants missing data on race, marital status, education, or income, 27,805 participants missing data on smoking, alcohol use, BMI, or prescription medication use, 105 participants missing depression screening scores based on the Patient Health Questionnaire-9 (PHQ-9) and 65 participants missing data on CVD. Participants with PHQ-9 scores ≥ 10 were defined as having depression. This cutoff is the standard clinical threshold for identifying moderate-to-severe depression, possessing high sensitivity and specificity for detecting Major Depressive Disorder (20), and is recommended for depression screening in cardiovascular populations (21). A baseline demographic characteristics table was constructed using the complete dataset with all covariates.

NHANES data from 2005–2020 were merged across seven survey cycles. Following NHANES analytic guidelines, variables were harmonized across cycles and combined interview sampling weights were constructed. For interview-based outcomes (MI and HF), we used the 2-year interview weight (WTINT2YR) for 2005–2016 and the subsample interview weight (WTINTPRP) for 2017–2020. To create multi-cycle pooled weights, each cycle-specific weight was divided by the total number of survey years (15.2 years).

To explore potential nonlinear relationships between depression and CVD risk, we applied restricted cubic spline (RCS) with 4 knots models. All analyses were performed using R software (version 4.3.2), with statistical significance defined as *p* < 0.05. Subsequently, weighted logistic regression models were used to evaluate associations between depression and HF, CAD, angina pectoris (AP), and MI, yielding odds ratio (OR) and 95% confidence interval (CI). Three hierarchical models were fitted using svyglm() with quasibinomial family. Variables were analyzed in three models: Model 1 (unadjusted), Model 2 (adjusted for demographic characteristics), and Model 3 (adjusted for all covariates). All models incorporated NHANES survey weights, strata (SDMVSTRA), and primary sampling units (SDMVPSU). OR and 95% CI were obtained by exponentiating model coefficients.

### 2.3 Public transcriptomic data preprocessing and differential expression analysis

Transcriptomic data were obtained from the GSE59867 human peripheral blood dataset. For MI, two comparisons were made: (1) patients on the first day of hospital admission (n = 111) vs. patients six months after discharge (n = 83); (2) patients on the first day of admission (n = 111) vs. patients with stable CAD and no history of MI (n = 46).

In addition, ST-segment elevation MI (STEMI) patients were divided into HF (n = 34) and non-HF (n = 30) groups based on plasma NT-proBNP levels and left ventricular ejection fraction parameters. MDD data were obtained from two human peripheral blood datasets: GSE38206 and GSE39653.

An additional validation dataset for MI (GSE62646) included 28 admission-day samples and 28 six-month follow-up samples. For HF validation, we utilized the GSE141910 dataset obtained from human left ventricular tissue, comprising 166 samples from patients with non-ischemic dilated cardiomyopathy (DCM) and 166 healthy controls. This dataset was selected to represent a chronic, non-ischemic HF phenotype, distinct from the acute ischemic MI samples. Probes were annotated with gene symbols. Probes with unmatched or multiple gene symbols were excluded, as were duplicate gene symbols.

Differential expression analysis was performed using the limma R package (V3.58.1) (22). Differentially expressed genes (DEGs) were defined as those with *p* < 0.05. For MI, only genes co-upregulated or co-downregulated across both comparisons were retained. Visualizations were generated using the ggplot2 (V3.5.2) and pheatmap (V1.0.12) R packages, including volcano plots and heatmaps. Subsequent downstream analyses were based on the first MI dataset.

We collected transcriptome sequencing data containing MDD patients and healthy donors (GSE38206 and GSE39653) to define MDD-related genes. Batch effects among the two datasets were corrected using the ComBat function from the “sva” R package with default parameters, specifying the batch variable as the dataset ID. Then the effectiveness of batch correction was assessed with principal component analysis (PCA) plots.

### 2.4 RNA-seq library preparation and sequencing

Total RNA was extracted from peripheral blood monocytes of 14 subjects, including 6 MI patients and 8 non-MI controls, and quantified using a NanoDrop spectrophotometer. mRNA was isolated via polyA selection and fragmented, and double-stranded cDNA was synthesized using random hexamer primers. The cDNA underwent end-repair, phosphorylation, and adapter ligation, followed by size selection for 300 bp fragments and PCR amplification. The sequencing library was quantified with Qubit 4.0 and sequenced on the NovaSeq X Plus platform (PE150) using the NovaSeq Reagent Kit.

### 2.5 Analysis of bulk RNA-seq data

The quality of raw sequencing data was evaluated using FASTQC (http://www.bioinformatics.babraham.ac.uk/projects/fastqc/). Adapter sequences and low-quality reads were removed using NGS QC toolkits. The trimmed reads were aligned to the human reference genome (hg19) using HISAT2 version 2.2.1 with default parameters. Uniquely mapped reads were used as input for Stringtie to quantify gene expression levels as transcripts per million. Differential gene expression analysis was conducted using the DESeq2 package in R.

### 2.6 Machine learning identification and validation of core MDD-related genes

We first applied LASSO regression to select the most informative biomarkers among MDD-mediated CVD genes. Genes screened from MI and HF were intersected, and the overlapping genes were subjected to LASSO regression. The common genes filtered from both MI and HF were then further analyzed using LASSO regression, and diagnostic models with nonzero coefficients were constructed with the R package “glmnet” (v4.1.8). Model parameters were set to 10-fold cross-validation (cv.glmnet) to determine the optimal regularization parameter (λ). Risk scores (RS) were calculated based on the regression coefficients of CVD-related MDD genes, and the risk of developing CVD was defined as follows:


RS−MI=(−5.6364)×TMEM43+(−6.4226)×C1orf174+(−1.1979)×L3MBTL4+(−0.0007)×OR52N4+4.2521×SLC25A20+1.2958×MISP3

RS−HF=9.2409×TMEM43+(−5.8384)×C1orf174+3.5975×L3MBTL4+(−2.6360)×OR52N4+2.6426×SLC25A20+3.8850×MISP3

PCA was used to reduce dimensionality and to distinguish differences in RS between healthy and CVD samples. Receiver operating characteristic (ROC) curves were plotted to evaluate the classification performance of the models.

To evaluate the robustness of the identified biomarkers, we performed a stability analysis using bootstrap resampling. Specifically, we generated 1,000 bootstrap samples by random sampling with replacement from the original dataset. LASSO regression was applied to each bootstrap sample, and the selection frequency of each gene was recorded. Genes that were selected in more than 70% of the iterations were considered robust diagnostic markers. This procedure ensures that the identified gene signature is not an artifact of random data partitioning and reflects consistent biological relevance.

To further strictly evaluate the robustness of the diagnostic model and rule out potential overfitting, we performed a repeated cross-validation analysis on the discovery datasets. Specifically, we repeated the cross-validation process 50 times to generate a distribution of AUC values. This approach allows for the estimation of the mean AUC and its 95% confidence interval (CI), providing a more reliable assessment of the model’s stability compared to a single data split.

### 2.7 Immune microenvironment and MDD correlation analysis

Immune cell infiltration was estimated for 22 immune cell types using the CIBERSORT algorithm (23). Immune pathway activity levels were evaluated by single-sample gene set enrichment analysis (ssGSEA), with immune-related gene sets obtained from the ImmPort database (http://www.immport.org) (24). The GSVA R package (V2.1.2) was used to convert individual gene expression matrices into pathway-level expression scores (25). Wilcoxon tests were used to compare immune cell proportions, immune pathway enrichment scores, and expression levels of human leukocyte antigen (HLA) family genes between healthy and CVD samples. Spearman correlation analyses were conducted to assess associations among immune cell fractions, immune pathway enrichment scores, HLA gene expression, and MDD gene expression. Spearman’s rank correlation coefficient (r) was used to quantify the associations between the 184 MDD-related genes and 22 immune cell subsets (estimated by CIBERSORT with 1000 permutations) with statistical significance defined as p < 0.05. Additionally, MI and HF samples were stratified into “high MDD-gene expression” and “low MDD-gene expression” groups based on the median expression of the 184 MDD-related genes. Wilcoxon tests were applied to compare immune cell proportions and pathway activity scores between the two groups. Correlation maps were visualized using the corrplot R package (V0.95).

### 2.8 Identification of MDD expression patterns

To explore similarities and differences in MDD expression patterns in cardiac (HF) and peripheral blood (MI) samples, we utilized the GSE141910 dataset (DCM tissue) for HF analysis. Due to platform-specific probe coverage, mapping the original 202 MDD-related candidates to this dataset resulted in 184 available genes. We employed this full intersection set for unsupervised clustering to preserve the comprehensive transcriptomic landscape of MDD-CVD comorbidity, ensuring that the identified endotypes reflect robust, systemic molecular patterns rather than single-gene fluctuations. These were subjected to unsupervised clustering using the ConsensusClusterPlus R package (V1.70.0) (26). The number of clusters (k) was set from 2 to 6, and clustering was repeated 1,000 times to ensure stability. The optimal cluster number was determined based on the cumulative distribution function (CDF) and the area under the CDF curve. PCA was then performed to validate clustering results. Wilcoxon tests were used to compare CVD-related MDD gene expression, immune cell fractions, immune pathway scores, and HLA gene expression across subtypes.

### 2.9 Functional enrichment analysis of the two MDD expression patterns

To further investigate the mediating role of MDD in CVD subtypes, DEGs between the two subtypes were identified using the “limma” package with a threshold of p < 0.05, and their intersection was defined as MDD mediated subtype-related genes. Gene Ontology-Biological Process (GO-BP) and Kyoto Encyclopedia of Genes and Genomes (KEGG) enrichment analyses were then performed using the “clusterProfiler” package to reveal the biological characteristics of these genes. To capture the biological changes occurring within each CVD subtype, the gene sets “h.all.v2025.1.Hs.symbols” and “c2.cp.kegg_legacy.v2025.1.Hs.symbols” were downloaded from MSigDB (https://www.gsea-msigdb.org/) (27). Subsequently, the GSVA algorithm was applied to quantify the activity of HALLMARK and KEGG pathways, followed by differential analysis using limma. Pathways with *p* < 0.05 were considered to show significant activity differences between the two subtypes.

## 3 Results

### 3.1 Causal effects of MHD on CVD in MR

**Figure 1:**
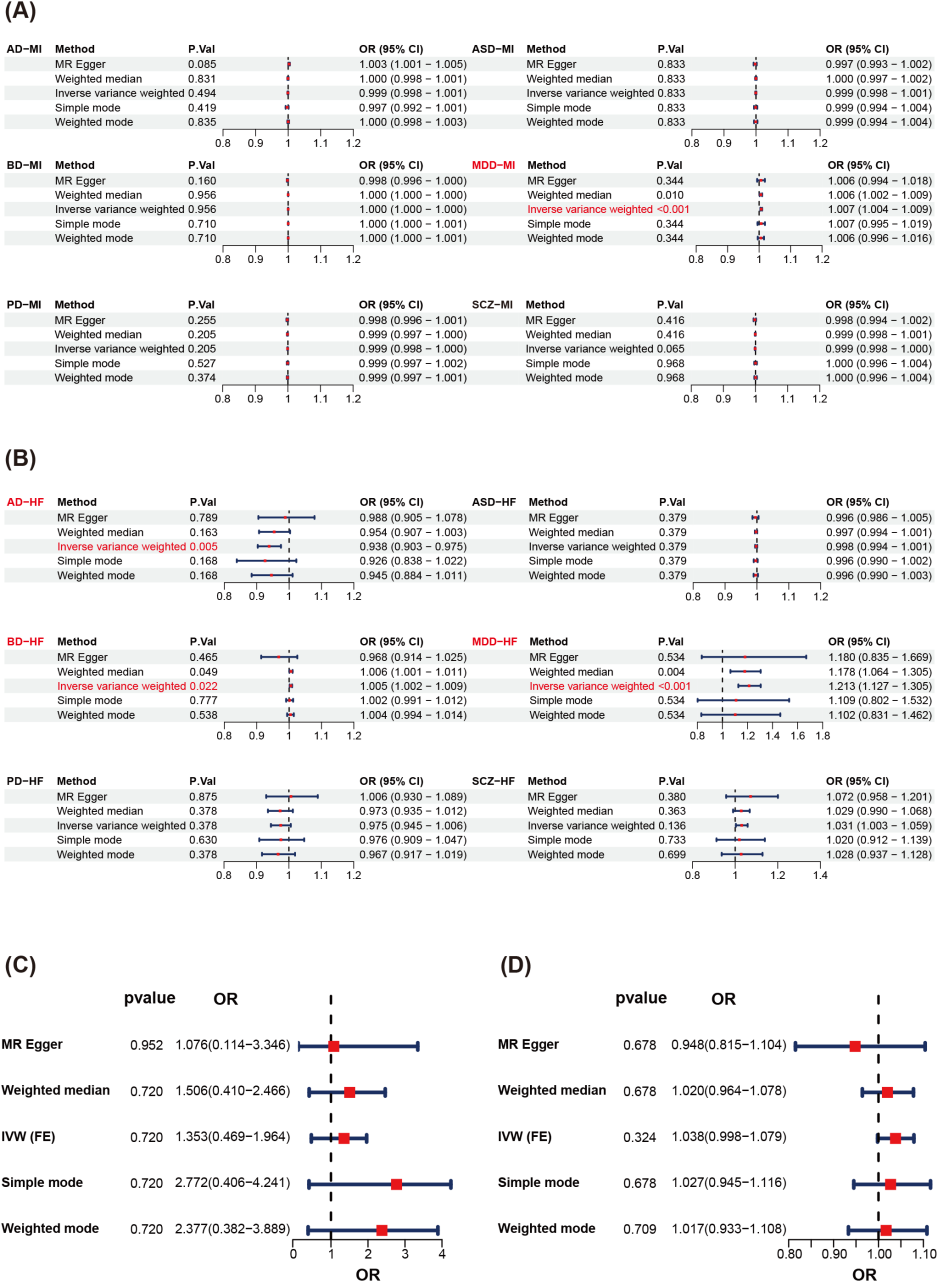
MR analysis of MHD and CVD. **(A)** Influence of MHD traits on MI by MR models. **(B)** Influence of MHD traits on HF by MR models. Effect estimates represent the change in outcome risk per unit increase in the log-odds of genetic liability to the exposure. **(C)** Reverse MR of MDD and MI. **(D)** Reverse MR of MDD and HF.

To investigate genome-wide associations between various MHD and multiple CVD, we performed two-sample MR analyses to test causal effects of six MHD (AD, ASD, BD, MDD, PD, SCZ) on two CVD outcomes (MI, HF). MR-PRESSO identified several outlier SNPs across different exposure–outcome pairs, which were removed to obtain corrected estimates. After applying FDR correction for 12 MR tests, we observed significant causal effects of MDD on MI and HF (p < 0.001). The effect sizes represent the risk change per unit increase in the log-odds of genetic liability to MDD. In addition, AD had a significant negative effect on HF, whereas BD showed a significant positive effect on HF (**Figures 1A, B**). We then performed reverse MR analyses for MDD and CVD to assess potential reverse causality; no significant reverse causal effects were detected (*p* > 0.05), excluding reverse causation as a confounder for the primary causal inference (**Figures 1C, D**, **Supplementary Tables S2**, **S3**).

However, for the association between schizophrenia (SCZ) and heart failure (HF), the MR-PRESSO Global Test remained significant (P = 0.031) even after outlier removal, indicating persistent horizontal pleiotropy. Therefore, this association should be interpreted with caution and was not considered as evidence of a reliable causal effect.

### 3.2 Clinical association between depression and CVD

To further examine clinical associations between depression and CVD, we analyzed 11004 participants from the NHANES database, including 4,124 male (37.5%) and 6,880 female (62.5%). Participants were divided into non-depressed (n = 1,0083) and depressed (n = 921) groups based on PHQ-9 scores. Baseline characteristics of the participants are presented in **Table 1**. Across all variables analyzed, there were statistically significant differences between the non-depressed and depressed groups (*p* < 0.05), except for the prevalence of AP and the alcohol consumption.

**Table 1:** The characteristics of included participants.

Characteristic	N	Overall	No	Yes	P-value
Gender	11004				<0.001
Female		6,880 (62.5%)	6,200 (90.1%)	680 (9.9%)	
Male		4,124 (37.5%)	3,883 (94.2%)	241 (5.8%)	
Race/Ethnicity	11004				0.013
Mexican American		1,636 (14.9%)	1,492 (91.2%)	144 (8.8%)	
Non-Hispanic Black		2,745 (24.9%)	2,521 (91.8%)	224 (8.2%)	
Non-Hispanic White		3,783 (34.4%)	3,460 (91.5%)	323 (8.5%)	
Other Hispanic		1,124 (10.2%)	983 (87.5%)	141 (12.5%)	
Other Race		1,716 (15.6%)	1,627 (94.8%)	89 (5.2%)	
Educational level	11004				<0.001
High school graduate		2,486 (22.6%)	2,264 (91.1%)	222 (8.9%)	
Less than high school		2,457 (22.3%)	2,143 (87.2%)	314 (12.8%)	
More than high school		6,061 (55.1%)	5,676 (93.6%)	385 (6.4%)	
Income level	11004				<0.001
low income		5,403 (49.1%)	4,775 (88.4%)	628 (11.6%)	
middle/high income		5,601 (50.9%)	5,308 (94.8%)	293 (5.2%)	
Marital status	11004				<0.001
Formerly married		2,430 (22.1%)	2,131 (87.7%)	299 (12.3%)	
Married		6,474 (58.8%)	6,068 (93.7%)	406 (6.3%)	
Never married		2,100 (19.1%)	1,884 (89.7%)	216 (10.3%)	
Age (years)	11004				0.035
<40		3,704 (33.7%)	3,400 (91.8%)	304 (8.2%)	
>60		3,503 (31.8%)	3,252 (92.8%)	251 (7.2%)	
40-60		3,797 (34.5%)	3,431 (90.4%)	366 (9.6%)	
Lifestyle characteristics					
Smoking status	11004				<0.001
Current smokers		1,505 (13.7%)	1,267 (84.2%)	238 (15.8%)	
Former smokers		1,778 (16.2%)	1,622 (91.2%)	156 (8.8%)	
Non smokers		7,721 (70.2%)	7,194 (93.2%)	527 (6.8%)	
Drinking status	11004				0.126
Heavy drinkers		5,630 (51.2%)	5,090 (90.4%)	540 (9.6%)	
Moderate drinkers		1,044 (9.5%)	990 (94.8%)	54 (5.2%)	
Non drinkers		4,330 (39.3%)	4,003 (92.4%)	327 (7.6%)	
BMI	11004				<0.001
Normal weight		2,952 (26.8%)	2,756 (93.4%)	196 (6.6%)	
Obese		4,629 (42.1%)	4,146 (89.6%)	483 (10.4%)	
Overweight		3,423 (31.1%)	3,181 (92.9%)	242 (7.1%)	
Took antidepressent	11004	1,078 (9.8%)	798 (74.0%)	280 (26.0%)	<0.001
Cardiovascular diseases					
Heart failure	11004	322 (2.9%)	263 (81.7%)	59 (18.3%)	<0.001
Coronary heart disease	11004	378 (3.4%)	321 (84.9%)	57 (15.1%)	0.002
Angina pectoris	11004	231 (2.1%)	194 (84.0%)	37 (16.0%)	0.162
Myocardial infarction	11004	354 (3.2%)	309 (87.3%)	45 (12.7%)	0.006

**Figure 2:**
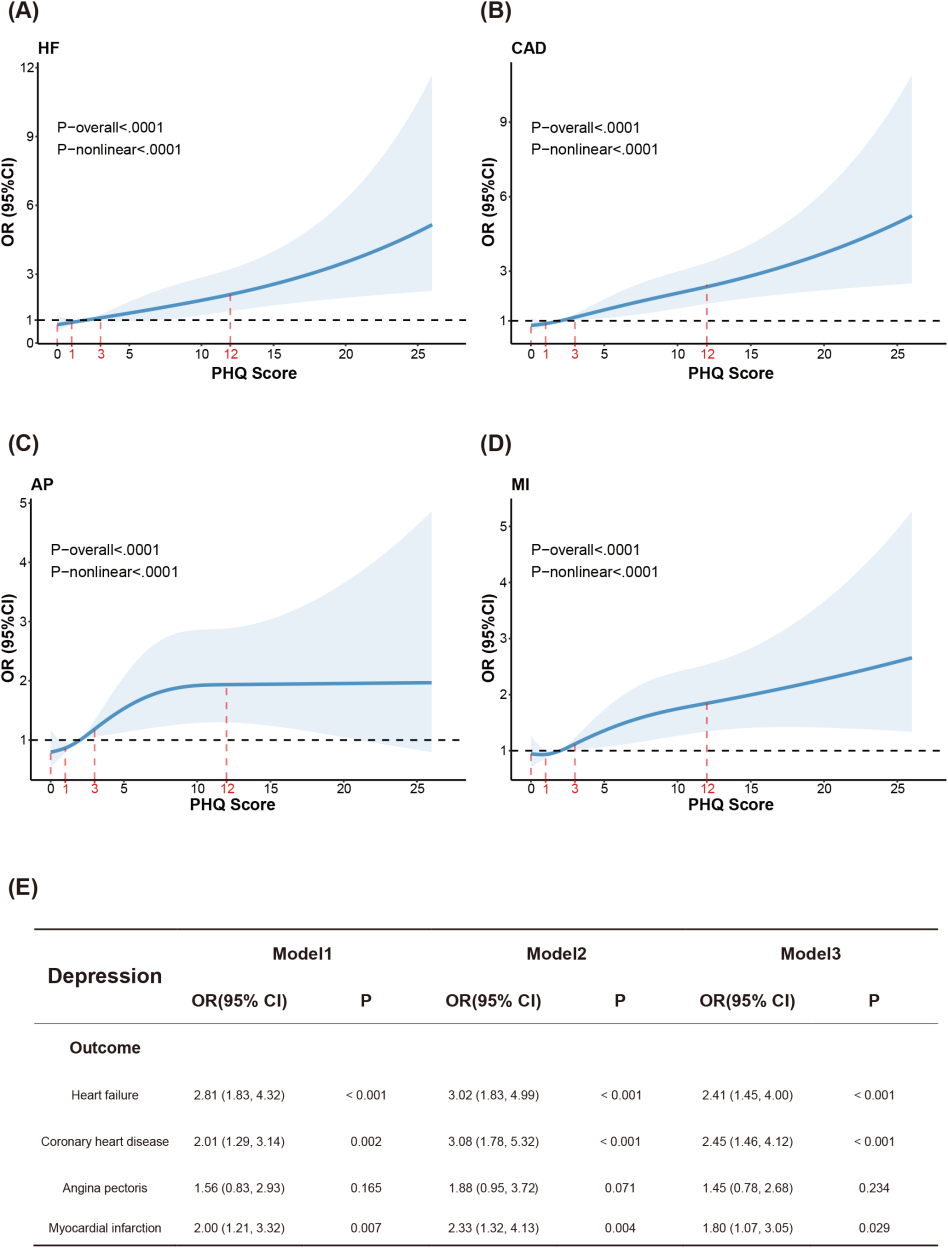
NHANES cohort analysis of depression and CVD. **(A)** RCS model of depression and HF. **(B)** RCS model of depression and CAD. **(C)** RCS model of depression and AP. **(D)** RCS model of depression and MI. **(E)** Logistic regression associations of depression with CVD in adults.

To explore potential nonlinear relationships and to evaluate the continuous association between depressive symptoms and CVD, we constructed RCS models with four knots to explore potential nonlinear relationships between depressive symptoms and CVD (**Figures 2A–D**). After adjusting for all covariates, PHQ-9 scores were positively and linearly associated with CVD risk; a highly significant nonlinearity was observed (*p* < 0.0001). The pointwise effect estimates with 95% CI are provided for detailed interpretation (**Supplementary Table S4**).

Multivariable logistic regression analyses were performed (**Figure 2E**). In the unadjusted model (Model 1), depression significantly increased the risk of all examined CVD outcomes except AP (p < 0.05), with the highest risk observed for HF (OR = 2.81, 95% CI: 1.83–4.32, p < 0.001). After adjustment for demographic characteristics (Model 2) and for all covariates (Model 3), these associations remained statistically significant.

### 3.3 Identification of MDD-related genes shared by distinct MI and HF phenotypes

**Figure 3:**
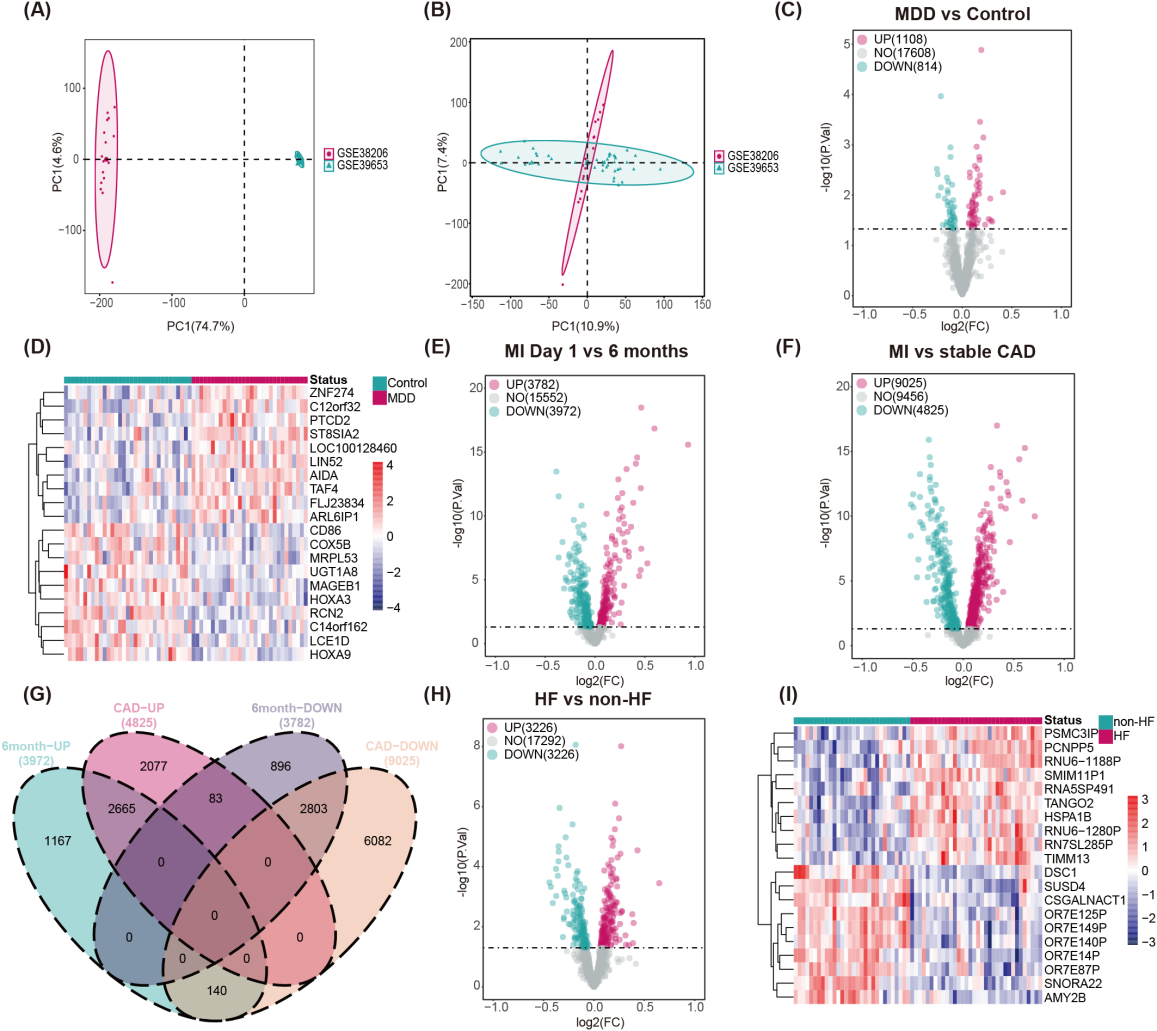
Identification of DEGs. **(A)** PCA of genes associated with healthy and MDD samples before batch effect removal. **(B)** PCA of genes associated with healthy and MDD samples after batch effect removal. **(C)** The volcano plot displays the expression changes of DEGs between healthy and MDD. **(D)** Heatmap shows 20 significantly dysregulated differentially expressed genes between healthy and MDD samples. **(E)** The volcano plot displays the expression changes of DEGs between MI patients at admission and at 6 months post-discharge. **(F)** The volcano plot displays the expression changes of DEGs between MI patients at admission and stable CAD patients without a history of MI. **(G)** Venn diagram shows the number of genes commonly upregulated and commonly downregulated between the two MI groups. **(H)** The volcano plot displays the expression changes of DEGs between HF and non-HF groups of STEMI patients. **(I)** Heatmap shows 20 significantly dysregulated differentially expressed genes between HF and non-HF groups of STEMI patients.

To explore molecular mechanisms by which MDD may affect the pathophysiology of both MI and HF, we collected transcriptome sequencing data containing MDD patients and healthy donors (GSE38206 and GSE39653) to define MDD-related genes. PCA revealed a substantial batch effect across datasets (**Figure 3A**). After batch correction, the integrated dataset consisted of 63 samples (30 MDD patients and 33 healthy donors) (**Figure 3B**). Compared with healthy donors, we identified 1,922 DEGs in MDD samples (*p* < 0.05), including 1,108 upregulated and 814 downregulated genes (**Figures 3C, D**, **Supplementary Table S5**).

To determine whether these MDD-related genes are consistently dysregulated in MI and HF contexts, we used the human peripheral blood dataset GSE59867. MI samples were analyzed in two comparisons: (1) patients on the first day of hospital admission (n = 111) vs. patients six months after discharge (n = 83); (2) patients on the first day of admission (n = 111) vs. patients with stable CAD and no history of MI (n = 46). For MI, DEGs common to both comparisons were merged, yielding 2,665 upregulated and 2,803 downregulated genes (**Figures 3E–G**, **Supplementary Table S6**). STEMI patients were also divided into HF (n = 34) and non-HF (n = 30) groups based on plasma NT-proBNP and left ventricular ejection fraction; differential analysis produced 6,014 DEGs (3,226 upregulated and 2,788 downregulated) (**Figures 3H, I**, **Supplementary Table S7**).

### 3.4 Six MDD marker genes effectively diagnose MI and HF

**Figure 4:**
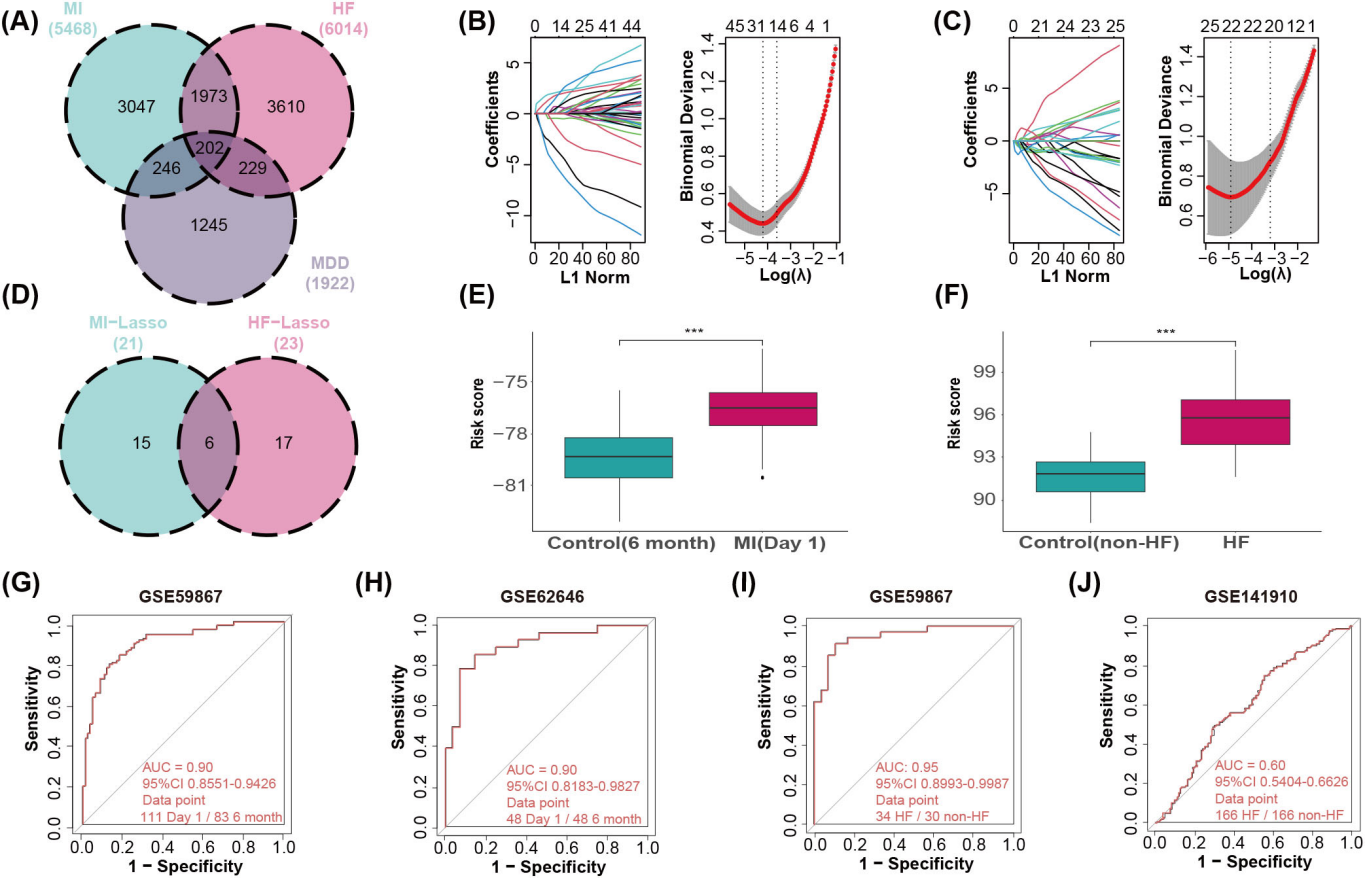
Identification of MDD-related genes in MI and HF through machine learning approaches. **(A)** Venn diagram shows 202 MDD-related genes commonly dysregulated in both MI and HF. **(B, C)** Parameter selection for the LASSO model in MI **(B)** and HF **(C)**. The left panels display the coefficient profiles, illustrating how coefficients of each gene shrink to zero as the L1 norm penalty increases. The right panels show the 10-fold cross-validation curves (binomial deviance vs. log(λ)). The vertical dotted lines mark the optimal λ values based on the minimum criteria (λ_min_) and the 1-standard-error criteria (λ_1se_). **(D)** Venn diagram shows the 6 MDD marker genes shared by MI and HF. **(E, F)** Boxplot comparing the risk scores of the LASSO model between controls, MI and HF. **(G, I)** ROC curve analysis of 6 MDD marker genes in MI and HF. **(H, J)** ROC curve analysis of the 6 MDD marker genes in the external validation cohorts of MI and HF.

To identify candidate MDD genes associated with diverse cardiovascular conditions (MI and HF), we first identified 202 MDD-related genes that were dysregulated in both MI and HF (**Figure 4A**). We then applied LASSO regression separately in MI and HF to select and reduce redundant MDD genes. As shown in the coefficient profile plots (**Figures 4B, C**, left panels), the LASSO algorithm shrinks the coefficients of less informative genes to zero as the penalty parameter (λ) increases, effectively distinguishing critical biomarkers from noise. To determine the optimal penalty, we performed 10-fold cross-validation (**Figures 4B, C**, right panels). The vertical dotted lines identify the λ value with the minimum deviance (λ_min_) and the value within one standard error (λ_1se_), balancing model accuracy and complexity. Using this method, we identified 21 and 23 MDD-related genes in MI and HF, respectively; six genes were shared by both sets (*TMEM43*, *C1orf174*, *L3MBTL4*, *OR52N4*, *SLC25A20*, *MISP3*) (**Figure 4D**, **Supplementary Table S8**). To further validate the reliability of these six genes, we conducted a bootstrap stability analysis with 1,000 iterations. The results demonstrated that all six genes were consistently selected with high frequency (Frequency > 70%) across the bootstrap samples (**Supplementary Figure S2**). This high selection probability confirms the robustness of the identified diagnostic signature and minimizes the likelihood of overfitting due to sample heterogeneity. Using the LASSO regression coefficients for these six shared marker genes, we constructed predictive models and calculated a RS for each sample (**Figures 4E, F**, **Supplementary Table S9**). As expected, disease samples showed significantly higher overall RS than healthy samples (*p* < 0.001). The RS predicted MI with an area under the ROC curve (AUC) of 0.90 (**Figure 4G**). The optimal Youden’s index (YI) was 0.66, corresponding to an RS cutoff of −77.6, with sensitivity = 0.79 and specificity = 0.87 (**Supplementary Table S10**). For HF, RS predicted disease with an AUC of 0.95 (**Figure 4I**); the optimal YI was 0.81, corresponding to an RS cutoff of 93.3, with sensitivity = 0.91 and specificity = 0.90 (**Supplementary Table S11**). We validated the MI model in an independent peripheral blood dataset (GSE62646; 28 admission-day samples and 28 six-month samples); results were similar (AUC = 0.90), indicating model robustness (**Figure 4H**). Although the initial LASSO model showed high diagnostic potential, we further assessed its stability through repeated cross-validation (50 iterations) on the discovery datasets. The model demonstrated consistent performance, with a mean cross-validated AUC of 0.887 (95% CI: 0.869–0.906) for MI and 0.907 (95% CI: 0.857–0.956) for HF (**Supplementary Figure S3**). These results confirm that the identified six-gene signature captures a robust biological signal rather than fitting random noise. However, validation of the HF model in a cardiac tissue dataset (GSE141910; 166 HF and 166 healthy samples) yielded poor performance (AUC = 0.60), suggesting that this risk prediction model, trained on blood-based signatures, may not fully capture the localized immune alterations in DCM-derived cardiac tissue samples (**Figure 4J**).

### 3.5 Independent validation of internal high-throughput RNA-seq cohorts

**Figure 5:**
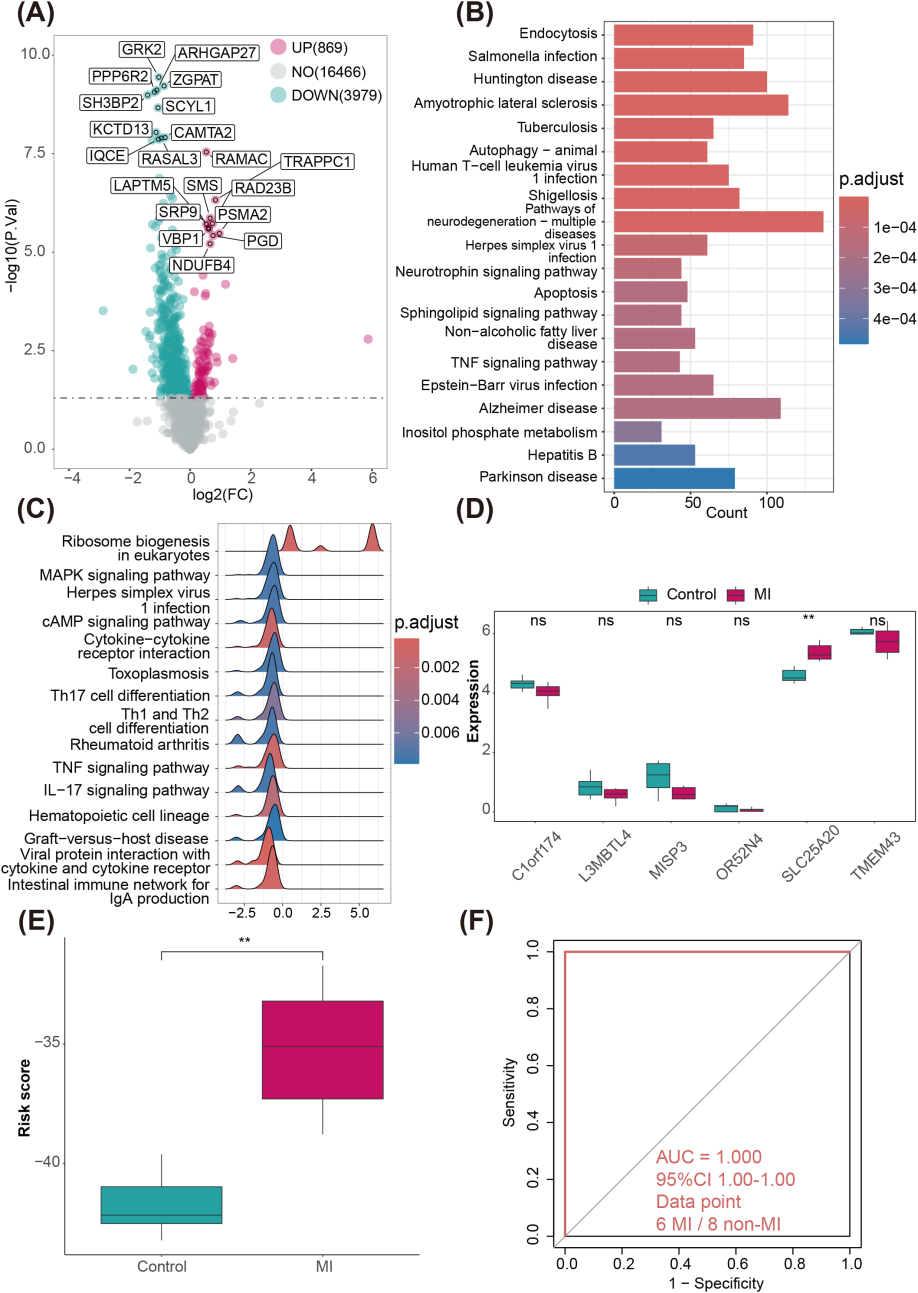
Independent transcriptomic validation and diagnostic evaluation of the RS model in MI. **(A)** Volcano and the heatmap plot of the DEGs from control and MI groups. **(B)** KEGG enrichment analyses based on the DEGs between control and MI groups. **(C)** GSEA analysis of KEGG pathways. **(D)** Box plots showing the expression of MDD marker genes in control and MI patients. **(E)** Box plot showing the distribution of risk scores between control and MI patients. **(F)** ROC curve analysis evaluating the diagnostic efficacy of the Risk Score (RS) model (trained on the GSE59867 discovery dataset) in the independent internal MI cohort.

Considering that both the training set and validation set used in this study were analyzed and validated based on microarray data, we generated and analyzed a high-throughput RNA-seq dataset of peripheral blood from 6 MI patients and 8 healthy donors to independently validate the transcriptomic features and diagnostic efficacy of the RS model. Transcriptomic profiling of the high-throughput cohort identified 4848 DEGs between healthy donors and MI (*p* < 0.05) (**Figure 5A**). KEGG pathway enrichment analysis showed significant enrichment of these DEGs in “Endocytosis”, “Salmonella infection” and “Huntington disease” (**Figure 5B**). Gene set enrichment analysis (GSEA) further confirmed significant enrichment in immune- and inflammation-related pathways, including the “TNF signaling pathway”, “IL-17 signaling pathway”, and “cytokine–cytokine receptor interaction” (**Figure 5C**). Core MDD-related genes displayed expression patterns in this cohort consistent with discovery datasets, with genes like *SLC25A20* notably upregulated in MI patients (**Figure 5D**). Additionally, the MI diagnostic model based on 6 MDD-related marker genes achieved an AUC of 1.00 in distinguishing MI patients from healthy controls in this independent RNA-seq cohort (**Figures 5E, F**). While the sample size of this validation cohort is limited (n = 14), this perfect classification aligns with the robust discriminatory ability demonstrated in our repeated cross-validation of the training set (**Supplementary Figure S3**). This suggests that the selected biomarkers have high generalizability across different platforms (microarray vs. RNA-seq) and distinct cohorts.

### 3.6 MDD marker genes are associated with immune microenvironment alterations in MI and HF

**Figure 6:**
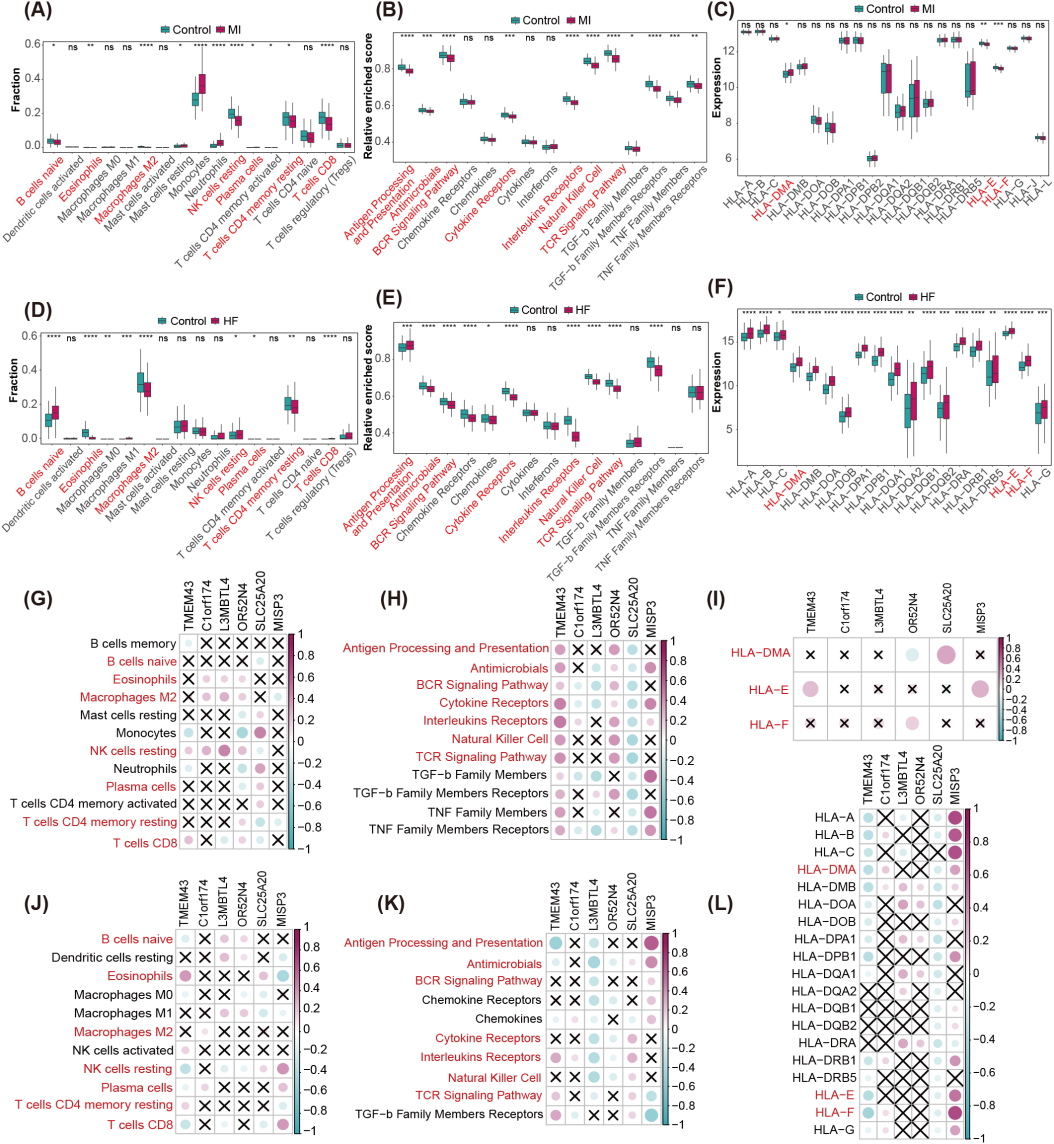
Analysis of the immune microenvironment in CVD and its correlation with MDD marker genes. **(A, B)** Differential abundance of infiltrating immune cells between control, MI, and HF groups was quantified using the CIBERSORT algorithm. **(C, D)** Differential enrichment of immune response gene sets between control, MI, and HF groups was analyzed using ssGSEA. **(E, F)** Comparison of HLA gene set expression levels between control, MI, and HF groups. **(G–L)** The dot-plot demonstrate the correlations between each dysregulated infiltrating immune cells, immune response gene sets and HLA gene, and each dysregulated autophagy gene. **(G, J)** Spearman correlation analysis of the six MDD marker genes with infiltrating immune cell abundance in MI **(G)** and HF **(J)**. **(H, K)** Spearman correlation analysis of the six MDD marker genes with immune response gene set scores in MI **(H)** and HF **(K)**. **(I, L)** Spearman correlation analysis of the six MDD marker genes with HLA family gene expression in MI **(I)** and HF **(L)**.

To further explore biological links between key MDD genes and immune microenvironment changes in MI and HF, we analyzed immune cell infiltration, immune response gene sets, and HLA family gene expression in MI and HF datasets. Immune cell subset infiltration was estimated using CIBERSORT, and differences were compared between MI and HF to identify commonalities and distinctions (**Supplementary Table S12**). Several immune cell types exhibited consistent change trends in both MI and HF—for example, eosinophils, M2 macrophages, plasma cells, and resting memory CD4+ T cells—suggesting these cells may play roles in shared immune mechanisms of MI and HF. Conversely, some immune cells showed opposite trends between the two diseases: naïve B cells, resting natural killer (NK) cells, and CD8+ T cells were significantly decreased in MI but showed no significant change or even increased levels in HF, which may reflect predominance of systemic acute inflammation in MI versus more chronic, localized cardiac immune regulation in HF (**Figures 6A, B**). We further compared enrichment differences of immune response gene sets between MI and HF (**Supplementary Table S13**). Many immune-related pathways showed concordant changes in both diseases—for example, antimicrobial response, B-cell receptors signaling, cytokine receptors signaling, interleukin receptors signaling, NK-cell related pathways, and T-cell receptors signaling were all significantly downregulated in MI and HF, suggesting suppression of these immune mechanisms in both conditions. However, some pathways displayed opposite trends: antigen processing and presentation was significantly upregulated in MI but significantly downregulated in HF, indicating differences in antigen recognition and processing mechanisms (**Figures 6C, D**). Analysis of HLA family gene expression (**Supplementary Table S14**) showed HLA-DMA upregulated in both MI and HF, with particularly high expression in HF. By contrast, HLA-E and HLA-F exhibited divergent expression patterns between the two diseases; these genes are important for immune regulation, especially NK cell and certain T cell activation (**Figures 6E, F**). Collectively, these differences suggest more prominent immune suppression or regulatory activation in HF, whereas MI is characterized predominantly by acute inflammation.

To bridge these findings with the identified MDD marker genes, we performed correlation analyses between these genes and the immune microenvironment components (**Figures 6G–L**). Regarding immune cell infiltration (**Figures 6G, J**), *C1orf174* was consistently positively correlated with M2 macrophages in both MI and HF, while *MISP3* showed a stable negative correlation with resting memory CD4+ T cells. Furthermore, both *L3MBTL4* and *SLC25A20* were significantly negatively associated with CD8+ T cells across both disease states.

Notably, a striking “polarity reversal” was observed in resting NK cells: *TMEM43*, *L3MBTL4*, and *OR52N4* were positively correlated with resting NK cells in MI but became negatively correlated in HF. Conversely, *SLC25A20* exhibited a negative correlation with resting NK cells in MI that flipped to a positive correlation in HF. Rather than a temporal transition, this likely reflects the distinct functional adaptation of NK cells within the acute systemic inflammatory environment of MI versus the chronic tissue remodeling microenvironment of DCM-derived HF.

In terms of immune response pathways (**Figures 6H, K**), several consistent regulatory patterns emerged. *MISP3* was positively correlated with the antimicrobial response in both datasets. In contrast, *L3MBTL4* showed consistent negative correlations with both the BCR signaling pathway and cytokine receptors signaling. Additionally, *TMEM43* and *C1orf174* were positively associated with interleukin receptors signaling, and *TMEM43* also maintained a positive correlation with TCR signaling in both MI and HF. These results suggest that these marker genes exert a conserved regulatory influence on specific immune cascades regardless of the pathological context.

Finally, the correlation with HLA family gene expression (**Figures 6I, L**) further highlighted the divergent regulatory logic between the two conditions. While *MISP3* was consistently positively correlated with HLA-E in both MI and HF, other key genes demonstrated significant reversals in their regulatory trends. Specifically, *SLC25A20* was positively correlated with HLA-DMA in MI but negatively correlated in HF. Similarly, *TMEM43* showed a positive association with HLA-E in MI that shifted to a negative correlation in HF. These inversions align with the distinct immune profiles of the two phenotypes, suggesting that *SLC25A20* and *TMEM43* may function as context-dependent molecular switches that respond differentially to acute ischemic stress (MI) and chronic non-ischemic remodeling (HF).

### 3.7 Distinct MDD-driven immune endotypes and their relationships with the immune microenvironment

**Figure 7:**
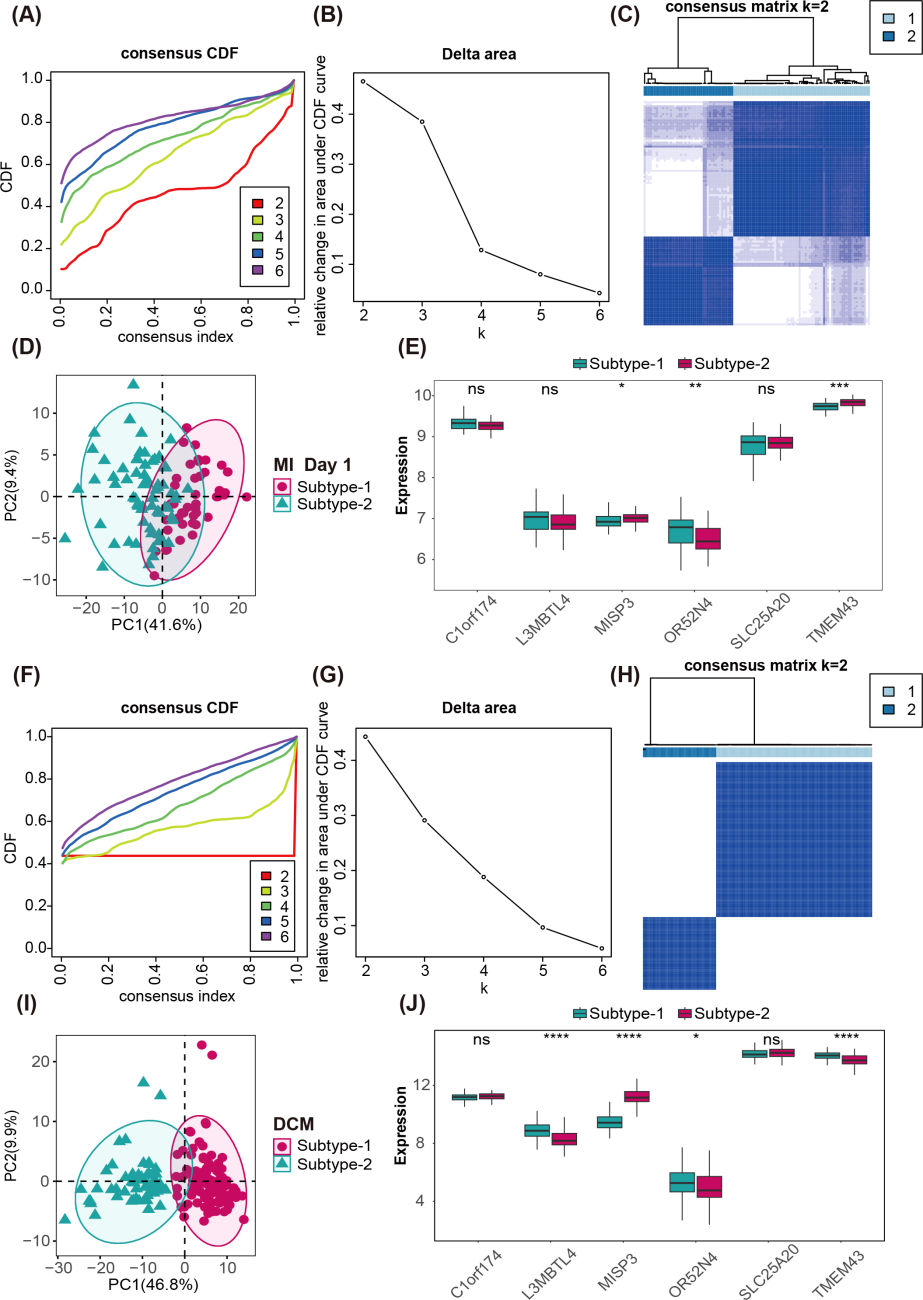
Identification of MDD-mediated patterns in CVD. **(A, F)** Cumulative distribution function curves of MI and HF for k = 2–6 clusters. **(B, G)** Relative change in the area under the Cumulative distribution function curve of MI and HF for k = 2–6 clusters. **(C, H)** Heatmap of the consensus matrices for MI and HF samples. **(D, I)** Principal component analysis of the transcriptomes under two MDD regulatory patterns in MI and HF, showing a clear distinction in transcriptomic features. **(E, J)** Box plots showing the expression differences of 6 MDD marker genes under different metabolic regulatory patterns in MI and HF.

To further investigate the similarities and differences in MDD expression patterns between HF and MI samples, we identified 184 MDD-related genes that were commonly associated with differential expression in HF and MI. Although not all these genes showed extremely significant expression changes individually, we argue that their collective variation reflects the full scope of MDD-mediated regulation—non-significant individual changes do not equate to lack of biological function. These genes collectively cover immune regulation, metabolic remodeling, and myocardial structural adaptation, which are integral to MDD’s role in CVD. Consensus unsupervised clustering based on expression of these 184 MDD genes was performed on MI and HF samples (**Supplementary Table S15**). MI samples were classified into two subtypes: subtype 1 (n = 44) and subtype 2 (n = 67) (**Figures 7A–C**, **Supplementary Table S16**). PCA demonstrated clear transcriptomic separation between the two MI subtypes (**Figure 7D**). Comparison of the six marker genes across the two MI subtypes showed that only three genes were significantly differentially expressed (**Figure 7E**), suggesting the remaining genes may not differentially regulate MI subtypes. HF samples were likewise clustered into two subtypes: subtype 1 (n = 113) and subtype 2 (n = 53) (**Figures 7F–H**, **Supplementary Table S17**). PCA indicated distinct separation between HF subtypes (**Figure 7I**). Comparison of the six marker genes across HF subtypes revealed the same three genes showing significant changes (**Figure 7J**), and notably expression trends of *OR52N4* and *MISP3* were opposite between MI and HF subtypes.

**Figure 8:**
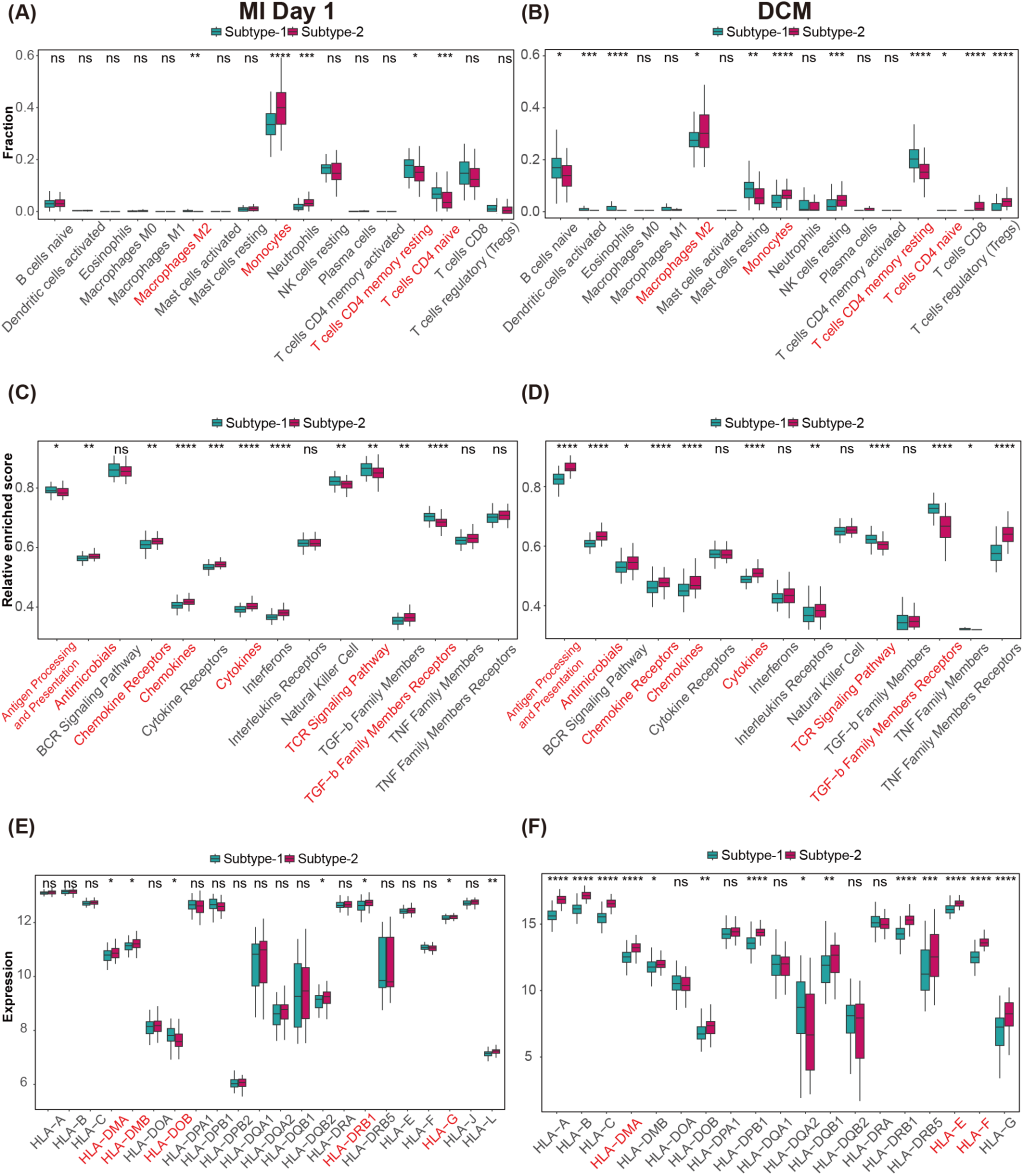
Differences in the immune microenvironment among CVD subtypes driven by MDD. **(A, B)** Differences in infiltrating immune cell abundance under two MDD regulatory patterns in MI and HF. **(C, D)** Differences in immune response gene set activity between the two MDD regulatory patterns in MI and HF. **(E, F)** Differences in HLA gene expression between the two MDD regulatory patterns in MI and HF.

To characterize shared and divergent immune microenvironment features between MI and HF subtypes, we evaluated infiltrating immune cells, immune response gene sets, and HLA family gene expression (**Supplementary Table S18**). Infiltrating immune cell analysis identified four cell types with significant differences common to both MI and HF; three showed concordant trends across subtypes—monocytes, resting memory CD4+ T cells, and naïve CD4+ T cells—whereas M2 macrophages exhibited opposite trends (**Figures 8A, B**). Regarding immune response pathways, seven pathways were commonly and significantly different between MI and HF subtypes; most followed the same directional changes (including antimicrobial response, chemokine receptor, chemokine, cytokine, TCR signaling, and TGF-β receptor family), whereas antigen processing and presentation displayed opposite trends (**Figures 8C, D**). For the HLA gene set, five HLA family genes were commonly dysregulated between MI and HF; HLA-DMA, HLA-DMB, HLA-DRB1 and HLA-G showed concordant changes, while HLA-DOB showed opposite trends (**Figures 8E, F**).

### 3.8 Biological functions underlying the CVD expression patterns

**Figure 9:**
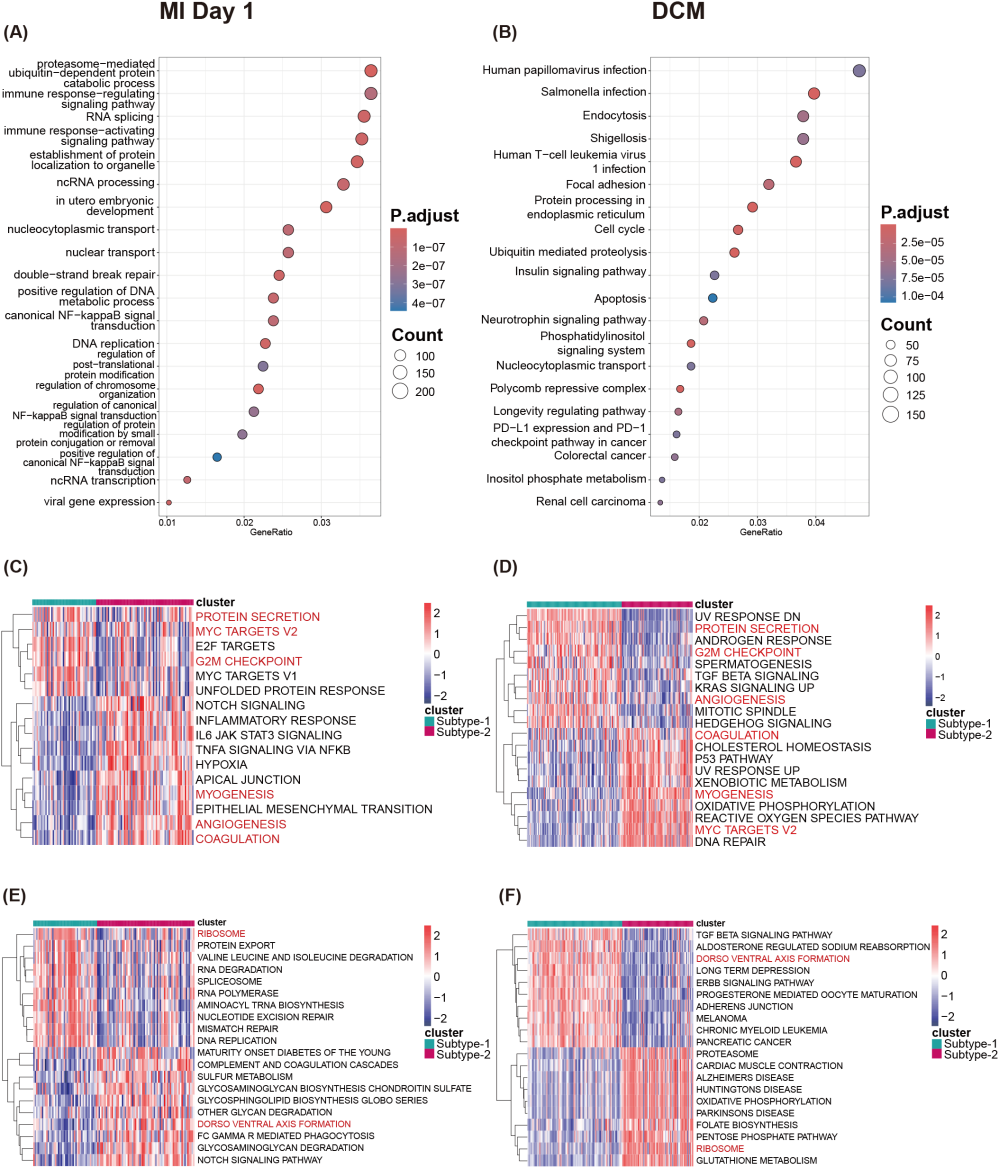
Biological functions underlying CVD expression patterns. **(A)** GO-BP enrichment analysis of genes shared across CVD subtypes. **(B)** KEGG enrichment analysis of genes shared across CVD subtypes. **(C, D)** The 16 most significantly different HALLMARKS pathways between two MDD-related expression patterns identified by GSVA analysis. **(E, F)** The 20 most significantly different KEGG pathways between two MDD-related expression patterns identified by GSVA analysis.

To further elucidate the role of MDD in immune regulation, we intersected 11,578 DEGs (**Supplementary Table S19**) between the two MI subtypes with 14,002 DEGs (**Supplementary Table S20**) between the two HF subtypes (*p* < 0.05) and identified 7,306 shared genes as MDD mediated subtype-related genes (**Supplementary Table S21**). Consistent with other transcriptomic studies that report shared immune-inflammatory genes between MDD and MI (e.g., *TLR2*, *ICAM1*, *S100A9*, *VCAN*) (28), these genes appeared among our MDD mediated subtype-related genes, suggesting possible immune-mediated cardiovascular damage. In agreement with these findings, GO-BP and KEGG enrichment analyses in our study showed significant enrichment of MDD mediated subtype-related genes in multiple immune-related pathways (e.g., NF-κB signaling) and protein degradation regulatory processes (e.g., ubiquitin-mediated proteolysis) (**Figures 9A, B**), further supporting a model in which MDD impacts MI and HF pathogenesis via regulation of immune responses and metabolic processes.

HALLMARK pathway analyses indicated that “G2M checkpoint” and “Protein secretion” were more active in subtype 1 of both MI and HF, whereas “Myogenesis” and “Coagulation” were active in subtype 2 of both diseases. We also observed pathways with opposing trends; for example, “MYC-version 2” was active in MI subtype 1 but in HF subtype 2, while “Angiogenesis” was active in MI subtype 2 but in HF subtype 1 (**Figures 9C, D**). Similarly, KEGG enrichment revealed pathways with inverse trends: “Ribosome” was active in MI subtype 1 and HF subtype 2, while “Dorso-ventral axis formation” was more prominent in MI subtype 2 and HF subtype 1 (**Figures 9E, F**).

## 4 Discussion

Our MR analyses suggest that genetic liability to MDD is associated directionally with higher risk of MI and HF, although modest effect sizes and residual pleiotropy cannot be fully excluded This finding is consistent with previous MR studies indicating that, at the genetic level, MDD exerts a unidirectional causal effect on CVD (10–12). In the NHANES clinical data, depression was associated with an increased incidence of CVD, and this association remained statistically significant after adjustment for available covariates. However, given the limited availability of psychiatric comorbidity data, this relationship should not be interpreted as fully independent. This is consistent with a previous NHANES cohort study (2007-2018), which emphasized the causal detrimental effects of MDD on CVD (10). Our study, however, spans a longer period (2005-2020) and further explores potential nonlinear relationships between depression and CVD risk. Notably, after adjusting for demographic and clinical variables, both MI and HF risks showed the similar upward trend, suggesting that MDD may influence these cardiovascular events through common pathological pathways.

Although depression was treated as an independent predictor in the NHANES analysis, several important psychiatric confounders—such as anxiety disorders, bipolar disorder, substance-use disorders, and full psychotropic medication use—could not be included due to NHANES data availability constraints. Specifically, anxiety and other mental health variables were only collected in a limited number of cycles (mainly 2007–2012), and detailed prescription medication information after 2017 is restricted-access and not available to us. Therefore, only antidepressant medication use, the only consistently available psychiatric treatment variable across 2005–2020, was included in the adjusted models. Consequently, residual confounding from unmeasured psychiatric comorbidities cannot be entirely excluded.

Multiple studies have suggested that MDD shares associated genes and signaling pathways with MI and HF, using diverse methods such as text mining (29), differential expression and pathway network enrichment analyses, and machine learning–based feature selection (30, 31). For MI, genes such as TLR2 and VCAN have been identified as MDD-related key genes (30, 31). For HF, STAT4 and COL1A2 have been identified as potential diagnostic biomarkers and therapeutic targets (32). In our study, we systematically mined MDD-related genes from two publicly available peripheral blood transcriptome datasets and found widespread expression abnormalities across both MI and HF datasets. Using differential analysis and LASSO feature selection, we identified six marker genes (*TMEM43*, *SLC25A20*, *C1orf174*, *L3MBTL4*, *OR52N4*, *MISP3*). Notably, *TMEM43* and *SLC25A20* have been reported to play important roles in myocardial energy metabolism and structural maintenance. Mutations in *TMEM43* are associated with hereditary cardiomyopathy and may affect myocardial remodeling (33), while *SLC25A20*, a mitochondrial carnitine transporter, is crucial for fatty acid metabolism, and its dysfunction may indirectly impair cardiac function (34). The discrepancy between the model’s performance in peripheral blood (AUC = 0.95) and cardiac tissue (AUC = 0.60) in HF suggests that these six biomarkers primarily reflect systemic immune-inflammatory dysregulation driven by MDD, rather than localized myocardial structural damage. This aligns with our findings that MDD remodels the immune microenvironment (e.g., M2 macrophages and T cell subsets), which can be detected in circulation.

However, this does not imply a lack of cardiac relevance. Among the identified genes, TMEM43 is critical for myocardial structural integrity, and SLC25A20 regulates mitochondrial fatty acid oxidation—a key metabolic process in heart failure. The detection of these metabolic and structural regulators in peripheral blood implies that MDD may induce a ‘systemic metabolic footprint’ that parallels cardiac pathophysiology. Thus, these peripheral signatures likely represent the neuro-immune-cardiac axis, serving as accessible indicators of the upstream inflammatory and metabolic burden placed on the heart by depression. In addition, the RS model exhibited excellent diagnostic performance in our independently generated high-throughput sequencing data. However, since the independent cohort collected in this study included a limited sample size (6 MI patients and 8 healthy individuals), our current results should be interpreted as hypothesis-generating. Although the model achieved high accuracy, the possibility of selection bias in small samples cannot be ruled out. Therefore, large-scale, multi-center prospective studies are required to further validate the universality and clinical utility of these biomarkers.

Previous studies have shown that both MI and HF exhibit extensive remodeling of the immune microenvironment, though with differences in pattern and intensity. MI is more prone to acute inflammatory responses, with significant upregulation of neutrophils and mast cells, highlighting their central roles in myocardial injury and repair (35). By contrast, HF is characterized by chronic inflammation and immune imbalance, with upregulation of dendritic cells and CD8+ T cells and downregulation of certain regulatory and reparative immune cells (e.g., M2 macrophages, eosinophils), reflecting sustained inflammatory activation and impaired immune function (36). Immune alterations in MDD have also been reported, including upregulation of monocytes and eosinophils, along with downregulation of resting CD4+ memory T cells and dendritic cells, suggesting immune dysregulation and impaired adaptive immune responses (37–39). These findings imply that MDD may amplify acute inflammatory responses in MI and exacerbate chronic immune imbalance in HF. In our study, we further compared the immune profiles of MI and HF and found that eosinophils, M2 macrophages, and resting CD4+ T cells displayed similar trends in both diseases, whereas naïve B cells, natural killer cells, and CD8+ T cells showed opposite changes. Moreover, we observed differential expression patterns in antigen processing and presentation pathways as well as key immune factors such as HLA-E and HLA-F between MI and HF, suggesting that MI is more associated with acute systemic inflammation, whereas HF is characterized by chronic immune dysregulation.

Consensus clustering demonstrated that MDD-associated transcriptomic signatures segregate into two reproducible immune endotypes that persist across acute myocardial infarction and chronic heart failure cohorts. These data indicate that MDD does not uniformly exacerbate cardiovascular disease through a single mechanistic axis; rather, it antecedently biases affected individuals toward one of two dichotomous immune configurations. The first configuration, designated the homeostatic/pro-fibrotic endotype, is enriched for ribosomal biogenesis and cell-cycle machinery, consistent with a compensatory reparative phenotype. The second, termed the inflammatory-metabolic endotype, exhibits pronounced activation of NF-κB and TNF-α signaling concomitant with broad metabolic dysregulation. The recurrence of these mutually exclusive transcriptional programs across divergent cardiovascular phenotypes establishes them as stable, MDD-mediated immunomodulatory imprints rather than transient stage-specific alterations of disease progression.

## 5 Conclusions

In summary, our study integrates genetic causality, clinical associations, transcriptional expression, immune mechanisms, and molecular subtyping, revealing robust MDD-driven immune endotypes that transcend specific cardiac pathologies. This study serves as a hypothesis-generating exploration, providing an original framework for future studies. While promising, these identified transcriptomic signatures and immune endotypes warrant further independent verification to establish their robustness as potential therapeutic targets.

## Data Availability

The original contributions presented in the study are publicly available. This data can be found here: https://ngdc.cncb.ac.cn/omix/release/OMIX012151.

## References

[1] W.H. Organization . World mental health report: Transforming mental health for all. Geneva, Switzerland: World Health Organization (2022).

[2] Vos T Lim SS Abbafati C Abbas KM Abbasi M Abbasifard M . Global burden of 369 diseases and injuries in 204 countries and territories, 1990–2019: a systematic analysis for the Global Burden of Disease Study 2019. Lancet. (2020) 396:1204–22. doi: 10.1016/S0140-6736(20)30925-9, PMID: 33069326 PMC7567026

[3] Roth GA Mensah GA Johnson CO Addolorato G Ammirati E Baddour LM . Global burden of cardiovascular diseases and risk factors, 1990–2019: update from the GBD 2019 study. J Am Coll Cardiol. (2020) 76:2982–3021. doi: 10.1016/j.jacc.2020.11.010, PMID: 33309175 PMC7755038

[4] Blazoski C Yao Z Bafna T Jelwan Y Burka S Blaha M . Mental health disorders and the development of cardiovascular disease: insights from the all of us research program. Am J Cardiol. (2025) 257, 31–37. doi: 10.1016/j.amjcard.2025.07.021, PMID: 40752773

[5] Parlati ALM Nardi E Basile C Paolillo S Marzano F Chirico A . Cardiovascular disease and psychiatric disorders: An-up-to date review. J Public Health Res. (2024) 13:22799036241278817. doi: 10.1177/22799036241278817, PMID: 39398345 PMC11468319

[6] Vigo D Thornicroft G Atun R . Estimating the true global burden of mental illness. Lancet Psychiatry. (2016) 3:171–8. doi: 10.1016/S2215-0366(15)00505-2, PMID: 26851330

[7] Depression W . Other common mental disorders: global health estimates. Geneva: World Health Organization (2017). p. 24.

[8] Kiryukhina SV Zhdanova YV Borisova AD Labunskiy DA Podsevatkin VG . Some pathogenic mechanisms of the development of mental disorders in patients with cardiology pathology. Med Biotechnol. (2025) 1:140–53. doi: 10.15507/3034-6231.001.202502.140-153

[9] Rymaszewska J Kiejna A Hadryś T . Depression and anxiety in coronary artery bypass grafting patients. Eur Psychiatry. (2003) 18:155–60. doi: 10.1016/S0924-9338(03)00052-X, PMID: 12814847

[10] Cai D Xia M Chen X Yagi K Xu L Wang B . Heartache and heartbreak: an observational and mendelian randomization study. Global Heart. (2024) 19:19. doi: 10.5334/gh.1302, PMID: 38371655 PMC10870952

[11] Lu Y Wang Z Georgakis MK Lin H Zheng L . Genetic liability to depression and risk of coronary artery disease, myocardial infarction, and other cardiovascular outcomes. J Am Heart Assoc. (2021) 10:e017986. doi: 10.1161/JAHA.120.017986, PMID: 33372528 PMC7955472

[12] Liu W Lin Q Fan Z Cui J Wu Y . Major depression disorder and heart failure: A two-sample bidirectional Mendelian randomization study. PLoS One. (2024) 19:e0304379. doi: 10.1371/journal.pone.0304379, PMID: 38809848 PMC11135699

[13] Haapakoski R Mathieu J Ebmeier KP Alenius H Kivimäki M . Cumulative meta-analysis of interleukins 6 and 1β, tumour necrosis factor α and C-reactive protein in patients with major depressive disorder. Brain behavior Immun. (2015) 49:206–15. doi: 10.1016/j.bbi.2015.06.001, PMID: 26065825 PMC4566946

[14] Gold PW . The organization of the stress system and its dysregulation in depressive illness. Mol Psychiatry. (2015) 20:32–47. doi: 10.1038/mp.2014.163, PMID: 25486982

[15] Liu H Luiten PG Eisel UL Dejongste MJ Schoemaker RG . Depression after myocardial infarction: TNF-α-induced alterations of the blood–brain barrier and its putative therapeutic implications. Neurosci Biobehav Rev. (2013) 37:561–72. doi: 10.1016/j.neubiorev.2013.02.004, PMID: 23415700

[16] Wang M Cheng L Gao Z Li J Ding Y Shi R . Investigation of the shared molecular mechanisms and hub genes between myocardial infarction and depression. Front Cardiovasc Med. (2023) 10:1203168. doi: 10.3389/fcvm.2023.1203168, PMID: 37547246 PMC10401437

[17] Hemani G Zheng J Elsworth B Wade KH Haberland V Baird D . The MR-Base platform supports systematic causal inference across the human phenome. elife. (2018) 7:e34408. doi: 10.7554/eLife.34408, PMID: 29846171 PMC5976434

[18] Bowden J Davey Smith G Burgess S . Mendelian randomization with invalid instruments: effect estimation and bias detection through Egger regression. Int J Epidemiol. (2015) 44:512–25. doi: 10.1093/ije/dyv080, PMID: 26050253 PMC4469799

[19] Bowden J Del Greco M F Minelli C Zhao Q Lawlor DA Sheehan NA . Improving the accuracy of two-sample summary-data Mendelian randomization: moving beyond the NOME assumption. Int J Epidemiol. (2019) 48:728–42. doi: 10.1093/ije/dyy258, PMID: 30561657 PMC6659376

[20] Kroenke K Spitzer RL Williams JB . The PHQ-9: validity of a brief depression severity measure. J Gen Internal Med. (2001) 16:606–13. doi: 10.1046/j.1525-1497.2001.016009606.x, PMID: 11556941 PMC1495268

[21] Lichtman JH Bigger JT Jr. Blumenthal JA Frasure-Smith N Kaufmann PG Lespérance F . Depression and coronary heart disease: recommendations for screening, referral, and treatment: a science advisory from the American Heart Association Prevention Committee of the Council on Cardiovascular Nursing, Council on Clinical Cardiology, Council on Epidemiology and Prevention, and Interdisciplinary Council on Quality of Care and Outcomes Research: endorsed by the American Psychiatric Association. Circulation. (2008) 118:1768–75. doi: 10.1161/CIRCULATIONAHA.108.190769, PMID: 18824640

[22] Ritchie ME Phipson B Wu D Hu Y Law CW Shi W . limma powers differential expression analyses for RNA-sequencing and microarray studies. Nucleic Acids Res. (2015) 43:e47–7. doi: 10.1093/nar/gkv007, PMID: 25605792 PMC4402510

[23] Newman AM Liu CL Green MR Gentles AJ Feng W Xu Y . Robust enumeration of cell subsets from tissue expression profiles. Nat Methods. (2015) 12:453–7. doi: 10.1038/nmeth.3337, PMID: 25822800 PMC4739640

[24] Bhattacharya S Andorf S Gomes L Dunn P Schaefer H Pontius J . ImmPort: disseminating data to the public for the future of immunology. Immunologic Res. (2014) 58:234–9. doi: 10.1007/s12026-014-8516-1, PMID: 24791905

[25] Hänzelmann S Castelo R Guinney J . GSVA: gene set variation analysis for microarray and RNA-seq data. BMC Bioinf. (2013) 14:7. doi: 10.1186/1471-2105-14-7, PMID: 23323831 PMC3618321

[26] Wilkerson MD Hayes DN . ConsensusClusterPlus: a class discovery tool with confidence assessments and item tracking. Bioinformatics. (2010) 26:1572–3. doi: 10.1093/bioinformatics/btq170, PMID: 20427518 PMC2881355

[27] Liberzon A Subramanian A Pinchback R Thorvaldsdóttir H Tamayo P Mesirov JP . Molecular signatures database (MSigDB) 3. 0. Bioinf. (2011) 27:1739–40. doi: 10.1093/bioinformatics/btr260, PMID: 21546393 PMC3106198

[28] You H Dong M . Identification of immuno-inflammation-related biomarkers for acute myocardial infarction based on bioinformatics. J Inflammation Res. (2023) 16:3283–302. doi: 10.2147/JIR.S421196, PMID: 37576155 PMC10417757

[29] Dai Z Li Q Yang G Wang Y Liu Y Zheng Z . Using literature-based discovery to identify candidate genes for the interaction between myocardial infarction and depression. BMC Med Genet. (2019) 20:104. doi: 10.1186/s12881-019-0841-8, PMID: 31185929 PMC6560897

[30] Yu C Zhang F Zhang L Li J Tang S Li X . A bioinformatics approach to identifying the biomarkers and pathogenesis of major depressive disorder combined with acute myocardial infarction. Am J Trans Res. (2023) 15:932., PMID: 36915729 PMC10006793

[31] Sun L Ren C Leng H Wang X Wang D Wang T . Peripheral blood mononuclear cell biomarkers for major depressive disorder: A transcriptomic approach. Depression Anxiety. (2024) 2024:1089236. doi: 10.1155/2024/1089236, PMID: 40226717 PMC11918809

[32] Huang K Zhang X Duan J Wang R Wu Z Yang C . STAT4 and COL1A2 are potential diagnostic biomarkers and therapeutic targets for heart failure comorbided with depression. Brain Res Bull. (2022) 184:68–75. doi: 10.1016/j.brainresbull.2022.03.014, PMID: 35367598

[33] Zink M Seewald A Rohrbach M Brodehl A Liedtke D Williams T . Altered expression of TMEM43 causes abnormal cardiac structure and function in zebrafish. Int J Mol Sci. (2022) 23:9530. doi: 10.3390/ijms23179530, PMID: 36076925 PMC9455580

[34] Pasquadibisceglie A Quadrotta V Polticelli F . In silico analysis of the structural dynamics and substrate recognition determinants of the human mitochondrial carnitine/acylcarnitine SLC25A20 transporter. Int J Mol Sci. (2023) 24:3946. doi: 10.3390/ijms24043946, PMID: 36835358 PMC9961348

[35] Dong H Yan S-B Li G-S Huang Z-G Li D-M Tang Y-L . Identification through machine learning of potential immune-related gene biomarkers associated with immune cell infiltration in myocardial infarction. BMC Cardiovasc Disord. (2023) 23:163. doi: 10.1186/s12872-023-03196-w, PMID: 36978012 PMC10052851

[36] Sun S Lu J Lai C Feng Z Sheng X Liu X . Transcriptome analysis uncovers the autophagy-mediated regulatory patterns of the immune microenvironment in dilated cardiomyopathy. J Cell Mol Med. (2022) 26:4101–12. doi: 10.1111/jcmm.17455, PMID: 35752958 PMC9279601

[37] Zhang J Xie S Chen Y Zhou X Zheng Z Yang L . Comprehensive analysis of endoplasmic reticulum stress and immune infiltration in major depressive disorder. Front Psychiatry. (2022) 13:1008124. doi: 10.3389/fpsyt.2022.1008124, PMID: 36353576 PMC9638134

[38] Huang S Li Y Shen J Liang W Li C . Identification of a diagnostic model and molecular subtypes of major depressive disorder based on endoplasmic reticulum stress-related genes. Front Psychiatry. (2023) 14:1168516. doi: 10.3389/fpsyt.2023.1168516, PMID: 37649561 PMC10464956

[39] Xiong S Liao L Chen M Gan Q . Identification and experimental validation of biomarkers associated with mitochondrial and programmed cell death in major depressive disorder. Front Psychiatry. (2025) 16:1564380. doi: 10.3389/fpsyt.2025.1564380, PMID: 40370590 PMC12075303

