# Supplementary material for: Mendelian randomization and transcriptome analysis reveal depression-driven regulatory patterns of the immune microenvironment in myocardial infarction and heart failure
Source: Front Immunol. 2026 Jan 29;17:1727699. doi: 10.3389/fimmu.2026.1727699 (PMC12894390; doi:10.3389/fimmu.2026.1727699)
Supplement: Supplementary file 2 [file Table2.docx]

Supplementary Material

# Supplementary Figures and Tables

## Supplementary Figures

**
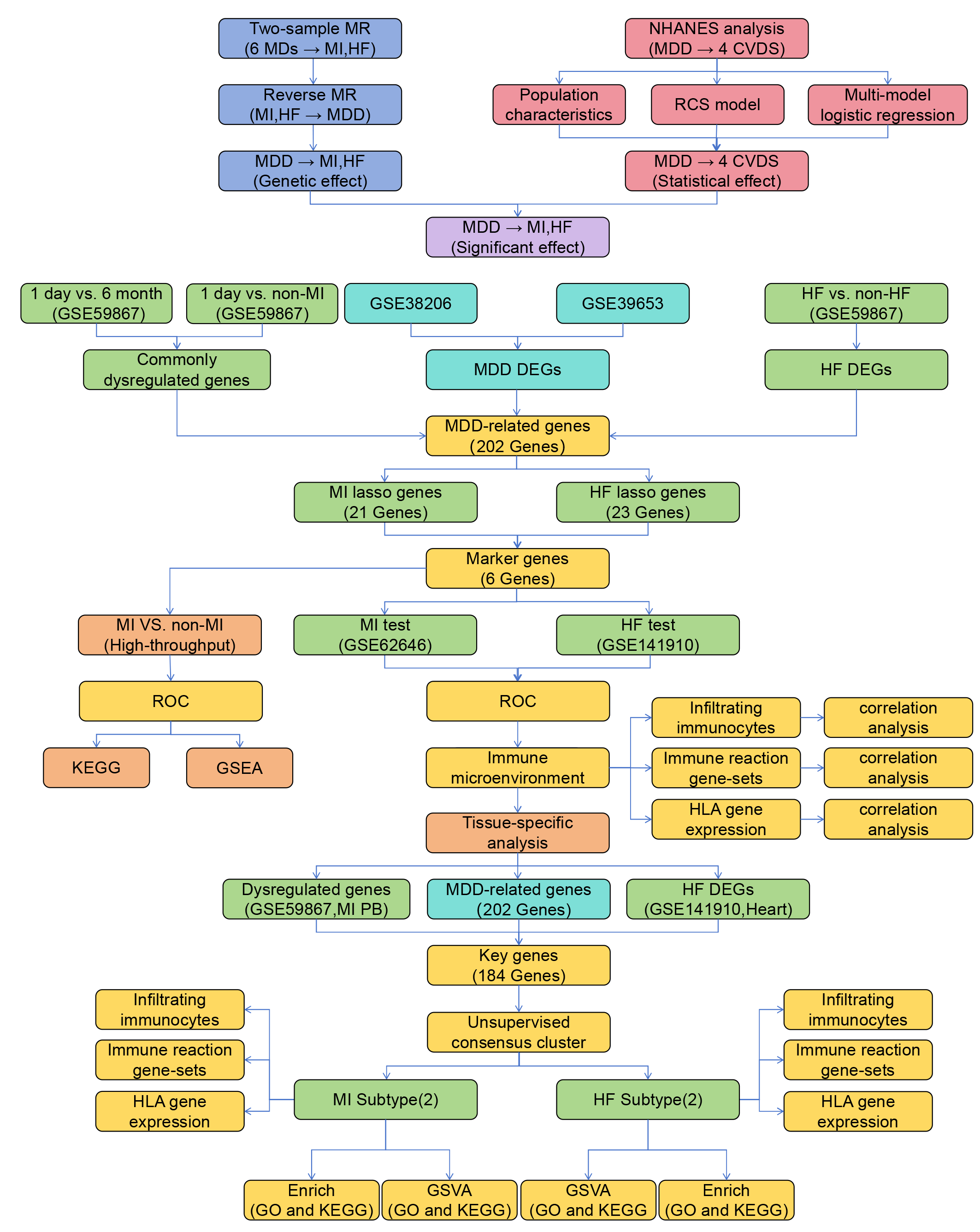
**

**Supplementary Figure 1.** Systematic framework integrating MR, NHANES, transcriptomic, and immune analyses of this study.


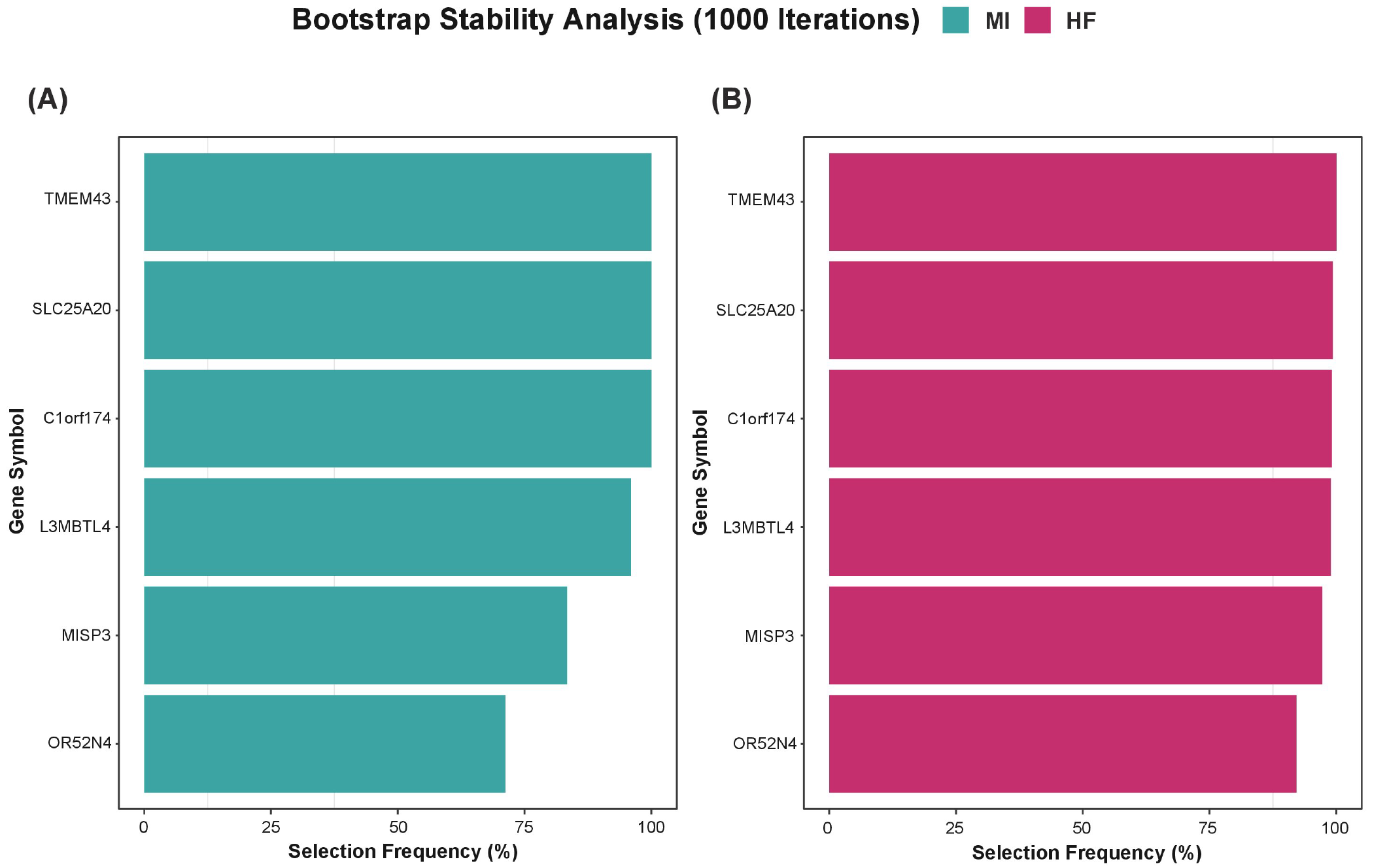


**Supplementary Figure 2.** Robustness validation of 6 MDD marker genes. **(A)** Selection frequency of the six diagnostic genes (*TMEM43*, *SLC25A20*, *C1orf174*, *L3MBTL4*, *OR52N4*, *MISP3*) in the MI dataset across 1,000 bootstrap iterations. **(B)** Selection frequency of the six diagnostic genes in the HF dataset across 1,000 bootstrap iterations.

**
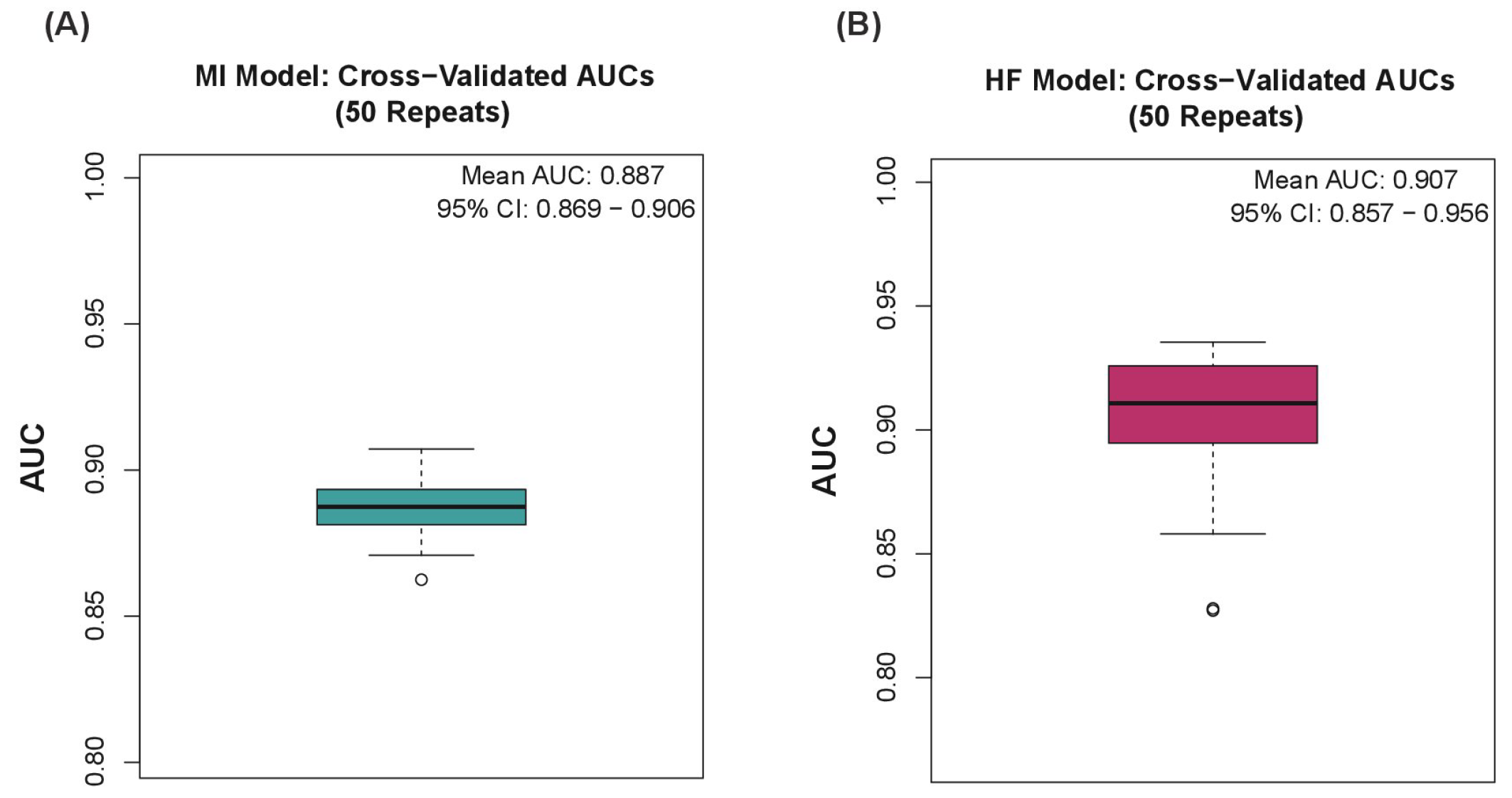
**

**Supplementary Figure 3.** Robustness validation of the diagnostic models in discovery datasets. **(A)** Repeated cross-validation (50 iterations) of the MI risk score model in the discovery dataset. **(B)** Repeated cross-validation of the HF risk score model in the discovery dataset.

## Supplementary Tables.

**Supplementary Table 1.** MR data source.

**Supplementary Table 2.** MR results for MI and MR-PRESSO analysis results.

**Supplementary Table 3.** MR results for HF and MR-PRESSO analysis results.

**Supplementary Table 4.** Specific effect estimates and confidence intervals for the RCS model of CVD.

**Supplementary Table 5.** Expression diversity of MDD.

**Supplementary Table 6.** Expression diversity of MI.

**Supplementary Table 7.** Expression diversity of HF.

**Supplementary Table 8.** The LASSO regression coefficient of MDD genes.

**Supplementary Table 9.** Risk scores of Control and CVD samples.

**Supplementary Table 10.** The performance of risk score in diagnosing MI in the training and testing sets.

**Supplementary Table 11.** The performance of risk score in diagnosing HF in the training and testing sets.

**Supplementary Table 12.** Diversity of immunocytes between Control and CVD samples.

**Supplementary Table 13.** Diversity of immune reaction gene-sets between Control and CVD samples.

**Supplementary Table 14.** Diversity of HLA gene expression between Control and CVD samples.

**Supplementary Table 15.** 184 genes shared by MI and HF.

**Supplementary Table 16.** The regulatory mode of MDD-mediated MI.

**Supplementary Table 17.** The regulatory mode of MDD-mediated HF.

**Supplementary Table 18.** Diversity of immune microenvironmental characteristics between regulatory modes of MDD-mediated CVD.

**Supplementary Table 19.** Differentially expressed MI genes between two MDD-mediated regulation patterns.

**Supplementary Table 20.** Differentially expressed HF genes between two MDD-mediated regulation patterns.

**Supplementary Table 21.** Common differentially expressed genes between MI and HF subtypes.
